# Identification of key proteins and pathways in cadmium tolerance of *Lactobacillus plantarum* strains by proteomic analysis

**DOI:** 10.1038/s41598-017-01180-x

**Published:** 2017-04-26

**Authors:** Qixiao Zhai, Yue Xiao, Jianxin Zhao, Fengwei Tian, Hao Zhang, Arjan Narbad, Wei Chen

**Affiliations:** 1 State Key Laboratory of Food Science and Technology, School of Food Science and Technology, Jiangnan University, Wuxi, Jiangsu People’s Republic of China; 2 UK-China Joint Centre on Probiotic Bacteria, Norwich, NR4 7UA UK; 3 Gut Health and Food Safety Programme, Institute of Food Research, Norwich, NR4 7UA United Kingdom; 4 Beijing Innovation Centre of Food Nutrition and Human Health, Beijing Technology & Business University, Beijing, 100048 People’s Republic of China

## Abstract

Our previous study confirmed the protective potential of *Lactobacillus plantarum* (*L*. *plantarum*) strains in alleviation of cadmium (Cd) toxicity *in vivo* and demonstrated that the observed protection largely depended on the tolerance of the strains to Cd-induced stress. It was also observed that there were significant intra-species differences in Cd tolerance of *L*. *plantarum* strains. In this study, we investigated the mechanism of Cd induced stress response of *L*. *plantarum* strains using the isobaric tags for relative and absolute quantitation (iTRAQ) based comparative proteomics. *L*. *plantarum* CCFM8610 (strongly resistant to Cd) and *L*. *plantarum* CCFM191 (sensitive to Cd) were selected as target strains, and their proteomic profiles in the presence and absence of Cd exposure were compared. We propose that the underlying mechanism of the exceptional Cd tolerance of CCFM8610 may be attributed to the following: (a) a specific energy-conservation survival mode; (b) mild induction of its cellular defense and repair system; (c) an enhanced biosynthesis of hydrophobic amino acids in response to Cd; (d) inherent superior Cd binding ability and effective cell wall biosynthesis ability; (e) a tight regulation on ion transport; (f) several key proteins, including prophage P2b protein 18, CadA, mntA and lp_3327.

## Introduction

Cadmium (Cd) is a representative non-essential element and known as an environmental hazard to human health. This heavy metal can contaminate the food chain and cause cumulative toxic effects in the liver, kidney, bone and the reproductive systems of humans^1–3^. Cd has also been classified as a potent human carcinogen by the International Agency for Research on Cancer^4^. Chelation therapies, the most commonly used treatments for heavy metal toxicity, have a number of safety and efficacy concerns and as yet none of them have been approved for clinical use against Cd poisoning in humans^5, 6^. The development of dietary supplements against Cd toxicity represents a potential alternative strategy.

Lactic acid bacteria (LAB) are members of commensal inhabitants of the human intestinal microbiota that can confer health benefits on the host^7^. Among these LAB, *Lactobacillus plantarum* (*L*. *plantarum*) strains have been widely used in the food industry as probiotics and functional food supplements^8^. Our previous studies have demonstrated that *L*. *plantarum* CCFM8610 can sequester Cd in the intestines of the host, which in turn promotes fecal Cd excretion and reduction in Cd accumulation in tissues, indicating that this strain can be considered a dietary supplement for the prevention and alleviation of Cd toxicity^9–11^. It was further noted that CCFM8610 offers significantly better protection than some other *L*. *plantarum* strains *in vivo* ^11^, and the protective effects were dependent on bacterial cell viability^9^.

Cd exposure, even at low levels, can disrupt cell wall synthesis, respiratory chain function and metal ion homeostasis in microorganisms, which in turn causes oxidative stress, DNA damage and energy metabolism dysfunctions, inducing extreme cellular toxic effects^12–14^. As *L*. *plantarum* cells contact with Cd directly in the intestines of the host, the protective effects of the strains against Cd toxicity largely depend on their abilities to thrive after Cd exposure. Therefore, understanding the mechanism of Cd tolerance of *L*. *plantarum* strains is essential for the development of probiotic based strategy against Cd toxicity. Our previous work showed that the minimum inhibitory concentration (MIC) of *L*. *plantarum* CCFM8610 (tested on Cd-containing agar plates) is more than 1000 mg/L, while some other *L*. *plantarum* strains have MIC values 10 to 20 times lower^15^. A bacterium is considered tolerant to Cd if its MIC value exceeds 100 mg/L^16^ or 112.4 mg/L of Cd^17^. Based on this definition, *L*. *plantarum* CCFM8610 can be considered a highly Cd-resistant strain. This may partly explain the protection of the host against Cd toxicity *in vivo* offered by this strain. Therefore, it is of interest to understand the resistance mechanism of *L*. *plantarum* strains against Cd exposure, and to explore why CCFM8610 is highly tolerant to Cd.

The physiological bases of heavy metal tolerance have been well investigated in environmental and industrial microorganisms such as *Pseudomonas* spp., *Escherichia coli*, *Bacillus subtilis* and *Saccharomyces cerevisia* ^12, 13, 18–20^. The related metabolomic pathways and key proteins identified include sulfur assimilation/glutathione synthesis pathway, metallothionein, oxidoreductase and ion transport proteins. However, comprehensive heavy metal tolerance mechanisms have not yet been well defined in LAB strains. To our knowledge, the Cd resistance modes have only been studied in *Streptococcus thermophiles* and *Lactococcus lactis*, with a focus on two Cd resistance-associated genes, CadA and CadC^21–24^. Numbers of reports also indicated the presence of these genes in some strains of *L*. *plantarum* species^25, 26^, but the Cd stress response network in these strains is yet to be elucidated.

Proteomics has been reported to be efficient to provide global physiological profiles of bacteria in protein level^27–29^. The most commonly used approaches for proteomic analysis are gel-based methods, such as two-dimensional gel electrophoresis (2-DE) and two-dimensional difference gel electrophoresis (2-D DIGE). However, these methods were reported to have limitations in sensibility, reproducibility, and proteome coverage^30, 31^. Isobaric tags for relative and absolute quantitation (iTRAQ) is a labelling approach that allows reliable quantitative description of differentially regulated proteins in complex systems. The main advantage is to have the possibility to analyze several samples together, with biological and technical replicates (4 or 8 plex). The subsequent use of high resolution mass spectrometry analyses provides accurate relative ratio between protein concentrations, present in the different samples^31^. Therefore, this approach was selected in this study.

In this study, the Cd-tolerance related key proteins and pathways within the *L*. *plantarum* species were investigated by using iTRAQ based proteomic approach. *L*. *plantarum* strains CCFM8610 (strongly resistant to Cd) and CCFM191 (sensitive to Cd) were selected for comparative proteomic analysis based on their differing Cd tolerant phenotype. The comparative proteomic profiles between non-stimulating and Cd-exposed conditions were compared in the two strains. The proteomic results were further confirmed by RT-qPCR and by the measurement of several biological properties of the bacterial cells in response to Cd exposure.

## Results

### Cd tolerance

**Figure 1:**
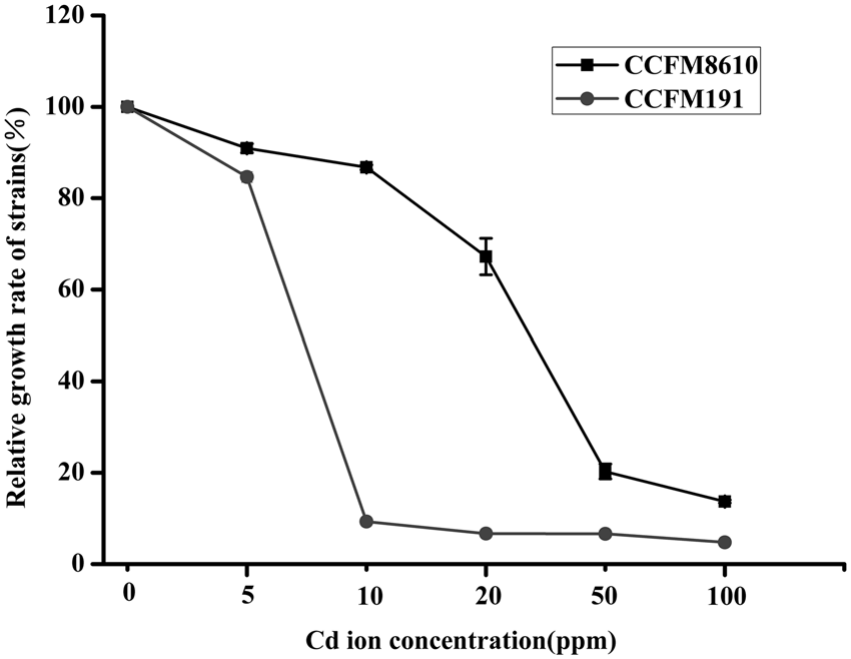
Relative growth rate of *L*. *plantarum* CCFM8610 and CCFM191 grown in MRS broth of different Cd concentrations. Relative growth rate was expressed as percentage of OD_600_ value of control culture (without Cd exposure) which was assigned a value of 100%. Data are expressed as mean ± SEM of three independent experiments per assay.

Twenty *L*. *plantarum* strains were cultured in MRS broth containing different concentrations of Cd, and the relative growth rates were determined (Table 1). While increasing Cd concentration caused a continuous decrease in the growth rate of all strains, significant Cd tolerance diversity could be observed, and the 20 strains could be categorized into four general groups, MIC > 50 mg/L, MIC = 50 mg/L, MIC = 20 mg/L and MIC = 10 mg/L. All tested strains showed no obvious growth since the Cd concentration reaching over 100 ppm (data not shown). *L*. *plantarum* CCFM8610 belonged to one of the eight strains with the highest MIC values, which was in agreement with our previous study on the tolerance of LAB strains tested on Cd-containing agar plates^15^. Based on the category of Cd tolerance of bacteria reported in a previous report^32^, CCFM191, one of the three strains with lowest MIC value (10 ppm), was selected as a Cd-sensitive strain for the comparative proteomic analysis with CCFM8610. As shown in Fig. 1, the dose of Cd at 5 mg/L (1/2 MIC value of the Cd-sensitive strain CCFM191) was selected for modeling a moderate and sublethal Cd exposure in the following experiments based on the previous related study^33, 34^.

**Table 1:** Relative growth rate of *L*. *plantarum* strains grown in MRS broth containing different Cd concentrations.

MIC value	Relative growth rate^a^ of strains grown in MRS broth containing different Cd concentrations (%)					
	Strains	5 ppm	10 ppm	20 ppm	50 ppm	100 ppm
MIC > 50	CCFM11	96.31 ± 0.55	91.52 ± 1.02	60.47 ± 0.72	18.98 ± 0.18	13.61 ± 0.47
	CCFM232	94.92 ± 0.40	89.36 ± 0.35	63.53 ± 3.24	16.38 ± 0.55	11.13 ± 0.17
	CCFM240	94.92 ± 0.34	92.49 ± 0.42	64.09 ± 1.31	17.62 ± 0.59	11.34 ± 0.23
	CCFM8610	90.92 ± 0.98	86.75 ± 0.54	67.21 ± 4.00	20.26 ± 1.63	13.71 ± 0.35
	CCFM405	101.93 ± 0.39	90.78 ± 2.26	60.30 ± 1.50	20.22 ± 0.90	11.61 ± 0.25
	CCFM595	102.01 ± 0.07	101.23 ± 0.23	91.38 ± 1.63	29.37 ± 0.88	10.52 ± 0.45
	CCFM579	96.81 ± 0.21	91.22 ± 0.17	64.47 ± 0.52	21.98 ± 0.03	12.88 ± 0.61
	CCFM8661	99.01 ± 0.59	92.20 ± 0.88	60.92 ± 0.28	19.44 ± 0.97	12.69 ± 0.31
MIC = 50	CCFM241	99.68 ± 0.09	100.04 ± 0.69	79.28 ± 2.49	8.73 ± 0.15	6.05 ± 0.30
	CCFM231	95.68 ± 2.11	92.13 ± 3.22	61.76 ± 2.74	7.61 ± 0.05	5.61 ± 0.09
	CCFM198	102.46 ± 0.30	94.91 ± 1.06	25.36 ± 3.71	7.50 ± 0.29	5.79 ± 0.14
	CCFM239	98.09 ± 0.51	95.67 ± 0.43	45.55 ± 2.47	7.56 ± 0.22	5.37 ± 0.14
MIC = 20	CCFM308	79.68 ± 0.51	19.30 ± 0.38	9.42 ± 0.33	6.00 ± 0.36	3.99 ± 0.28
	CCFM309	84.95 ± 1.46	21.58 ± 0.86	10.96 ± 0.43	6.42 ± 0.09	4.62 ± 0.11
	CCFM602	97.35 ± 0.62	84.03 ± 0.46	9.01 ± 0.56	4.55 ± 0.29	2.59 ± 0.08
	CCFM605	96.78 ± 0.45	72.04 ± 1.99	12.11 ± 0.67	4.32 ± 0.20	3.30 ± 0.45
	CCFM166	91.17 ± 2.12	37.30 ± 1.80	4.76 ± 0.47	3.20 ± 0.15	2.09 ± 0.03
MIC = 10	CCFM191	84.63 ± 1.14	9.30 ± 0.06	6.69 ± 0.25	6.64 ± 0.38	4.76 ± 0.10
	CCFM436	95.82 ± 0.84	13.94 ± 0.96	4.81 ± 0.02	4.16 ± 0.13	3.71 ± 0.01
	CCFM578	94.88 ± 0.48	9.51 ± 0.32	1.32 ± 0.09	4.56 ± 0.26	1.21 ± 0.25

^a^Relative growth rate of each strain was expressed as percentage of OD_600_ value of control culture (without Cd exposure) which was assigned a value of 100%. Data are expressed as mean ± SEM of three independent experiments per assay.

### Proteomic characteristics of two *L*. *plantarum* strains

**Figure 2:**
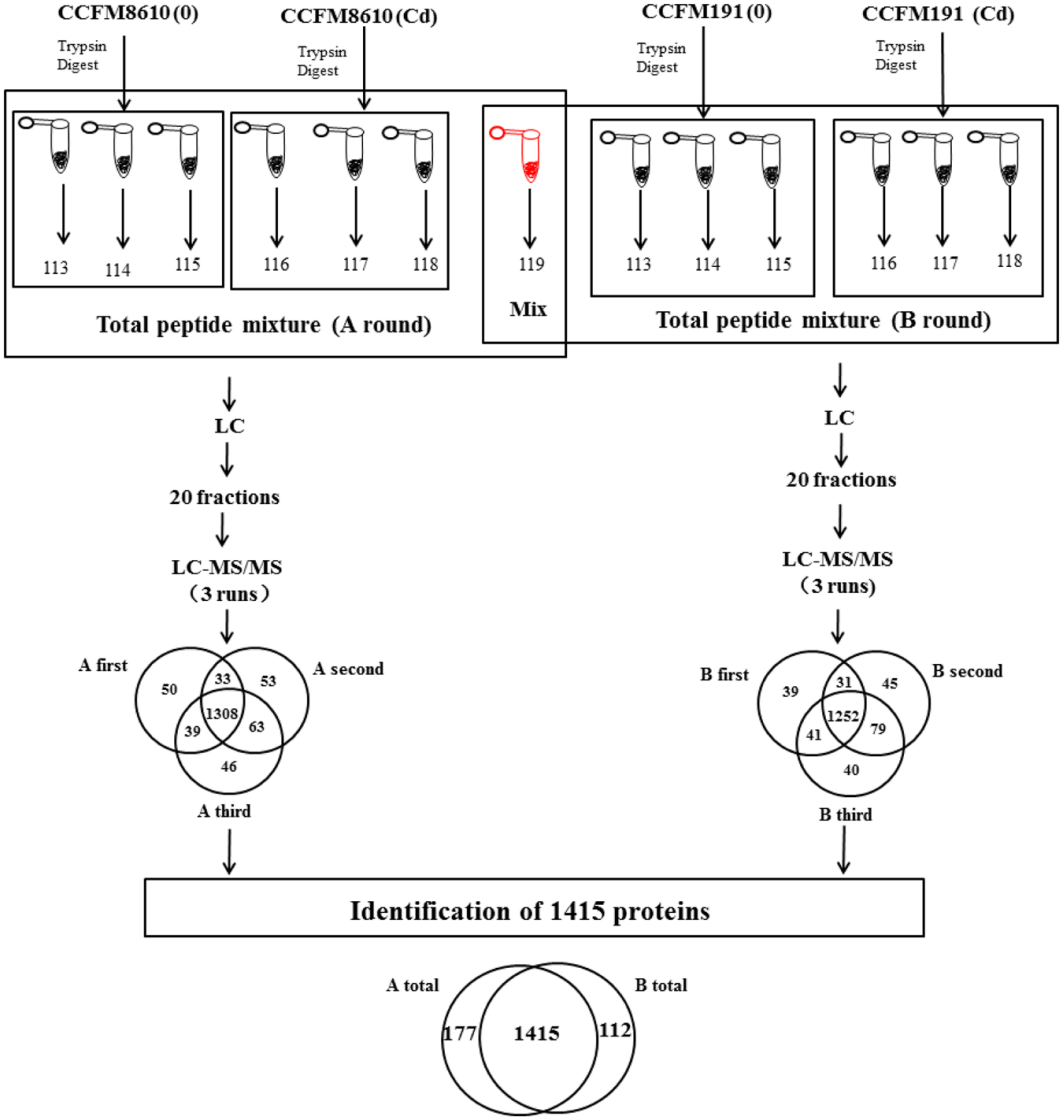
Workflow of iTRAQ experiment.

**Figure 3:**
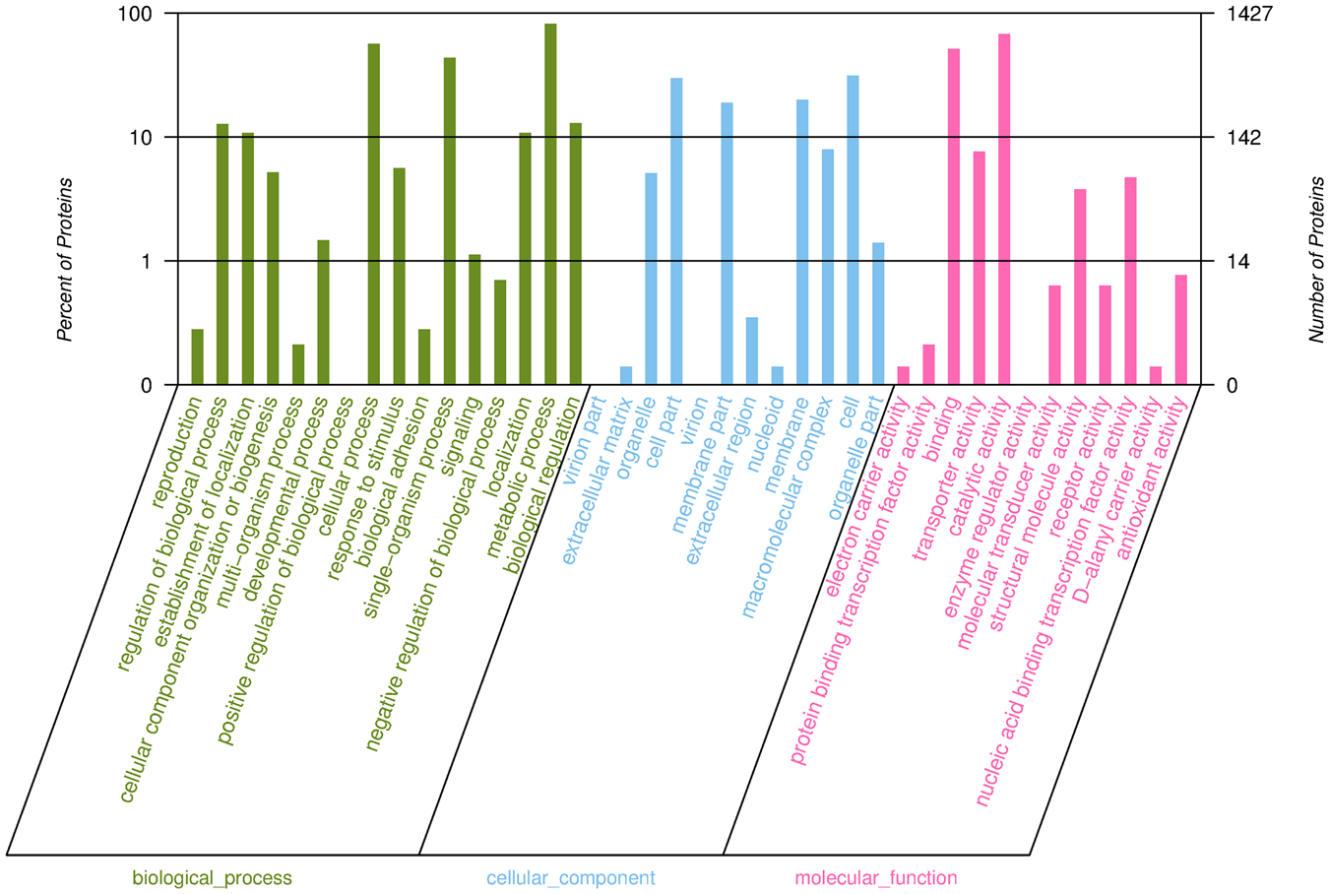
Functional categories of overlapped proteins identified in two *L*. *plantarum* strains by GO analysis. GO analysis was conducted by the software blast2go with the GO ID from the ensmbl database for each identified protein.

**Figure 4:**
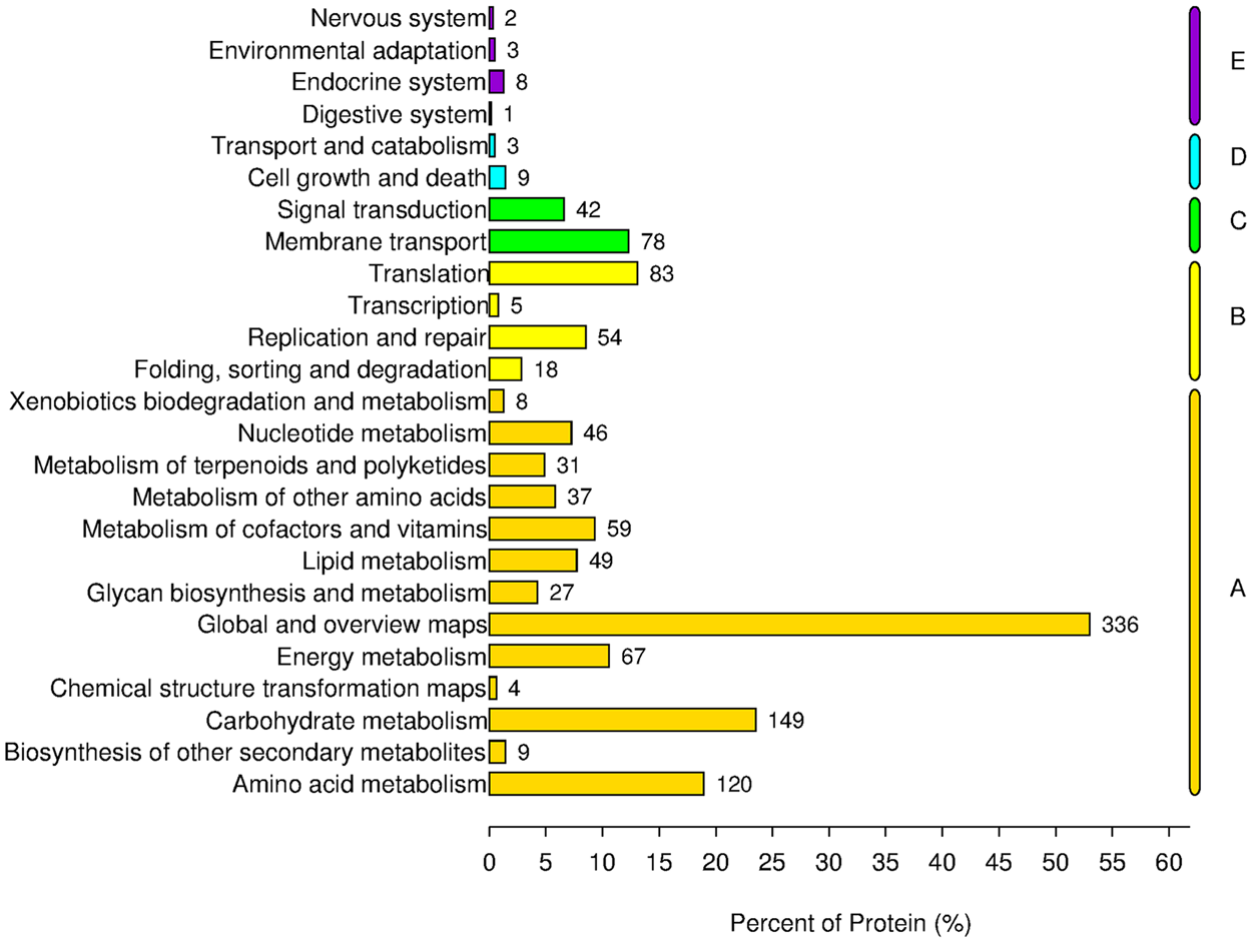
Related metabolomics pathways of overlapped proteins identified in two *L*. *plantarum* strains by KEGG classification. Each digital on the right of each bar indicates the number of proteins in each category. The letters A, B, C, D and E represent the 5 branches in KEGG pathways including Metabolism (A), Genetic Information Processing (B), Environmental Information Processing (C), Cellular Processes (D) and Organismal Systems (E).

1592 and 1527 proteins were detected and identified in CCFM8610 (with and without Cd exposure, “A” round) and CCFM191 (with and without Cd exposure, “B” round), respectively (Fig. 2). The 1415 overlapped proteins identified in both rounds were selected for protein function analysis. The protein function annotation was conducted by Gene Ontology (GO) analysis (Fig. 3), and the related metabolomics pathways of these proteins were analyzed by KEGG classification (Fig. 4). The results indicated that the detected proteins covered a large range with functions categorizing into biological process, cellular component and molecular function. The relevant metabolic pathways were related to carbohydrate metabolism (149 proteins), amino acid metabolism (120 proteins), translation (83 proteins), membrane transport (78 proteins), lipid metabolism (49 proteins), etc.

### Differentially expressed protein profiles

Intra-species difference of inherent proteomic profiles between Cd resistant and sensitive strains was evaluated, so as to pinpoint the proteins that might be implicated in the Cd stress response process. Total of 206 proteins with fold change more than 1.5 were selected as differentially expressed proteins (Table 2) for the comparison of CCFM8610 and CCFM191 in non-treated conditions (without Cd exposure). These proteins were categorized into global stress response, carbohydrate and lipid metabolism, transporters, some membrane and extracellular proteins, etc., based on the KEGG pathway analysis and their annotated functions in the Uniprot database. Compared with CCFM191, lower abundances of proteins that belong to global stress response, carbohydrate metabolism, phosphotransferase (PTS) system, two-component system, membrane protein and cell surface protein and hydrolase could be observed in CCFM8610. On the other hand, proteins involved in amino acid metabolism, nucleic acid metabolism and extracellular protein showed higher abundances in CCFM8610.

**Table 2:** Differentially expressed proteins between *L*. *plantarum* CCFM8610 and CCFM191 in Cd-free conditions.

Category^a^		Accession^b^	Description^c^	FC^d^
Global Stress Response	DNA repair, metabolism, regulation	F9UQY3	lp_2444; Prophage P2a protein 13	−3.30
		F9ULA4	lp_0641; Prophage P1 protein 18, DNA single-strand annealing protein RecT	−1.87
		Q6LWF5	traI; DNA topoisomerase	6.30
		F9URZ6	endA; DNA-entry nuclease	1.58
		Q88V16	mutS2; Endonuclease MutS2	−1.70
		Q88W97	recU; Holliday junction resolvase RecU	−1.52
	Oxidoreductase	F9UUA8	lp_3430; Peroxidase	5.14
		F9USL4	lp_3100; Aldo/keto reductase family protein	3.41
		F9UPP0	lp_1918; NAD(P)(H)-dependent oxidoreductase, quinone oxidoreductase (QOR) family	2.10
		F9URK6	lp_2732; NADPH-dependent FMN reductase family protein	−1.50
		F9UQD7	lp_2212; NADH-flavin reductase	−1.51
		F9UQI9	trxA2; Thioredoxin	−1.56
		F9UPR0	lp_1939; Oxidoreductase, medium chain dehydrogenases/reductase (MDR)/zinc-dependent alcohol dehydrogenase-like family	−1.64
		F9USA2	lp_2968; Nitroreductase	−1.65
		F9UTJ8	lp_3318; Aldo/keto reductase family protein	−1.65
		F9USK5	lp_3091; Short-chain dehydrogenase/oxidoreductase, atypical SDR family, subgroup 1	−2.83
		F9UT37	trxA1; Thioredoxin	2.04
		F9UTD6	lp_3236; Short-chain dehydrogenase/oxidoreductase, atypical SDR family, TMR-like	1.70
		F9URB2	lp_2604; NAD(P)-dependent oxidoreductase	1.53
		F9US17	nrdG; Anaerobic ribonucleoside-triphosphate reductase-activating protein	1.55
		F9UTT1	acdH; Acetaldehyde dehydrogenase	1.55
		F9UNI0	ribB; Riboflavin synthase, alpha chain	−5.89
		F9UR64	npr2; NADH peroxidase	−4.38
		F9ULD3	cat; Catalase	−3.76
		F9UN44	gshR2; glutathione reductase	−4.67
		F9UUC2	nox5; NADH oxidase	−2.30
		F9UTJ6	pflA; Pyruvate formate-lyase-activating enzyme	−2.09
		F9UUK7	lp_3545; D-arabitol-phosphate dehydrogenase	−3.08
		F9USK6	gabD; succinate-semialdehyde dehydrogenase (NAD(P) + )	−2.06
	Protein repair	F9UPH1	msrA; Peptide methionine sulfoxide reductase MsrA	−6.02
		Q88W33	msrB; Peptide methionine sulfoxide reductase MsrB	−2.67
	Protease	F9UTF5	lp_3259; Zinc-dependent proteinase	−1.96
		F9UT31	pepD1; Dipeptidase	−1.54
	Other	F9US30	lp_2952; Bacteriocin immunity protein	−1.58
		F9URC0	lp_2616; Bacteriocin immunity protein	−1.96
		F9USV1	hsp1; Small heat shock protein	−2.32
		Q88V03	ruvB; Holliday junction ATP-dependent DNA helicase RuvB	1.92
Regulation network	Transcriptional regulation	F9UT59	treR; Trehalose operon transcriptional repressor, GntR family	−1.91
		F9UM95	lp_0892; Transcription regulator, MarR family	−1.70
		Q88X36	argR1; Arginine regulator	−1.65
		F9UNC5	lp_1360; Transcription regulator, MarR family	2.06
		F9USP8	lp_3138; Bifunctional protein: transcriptional antiterminator, BglG family PTS system, EIIA component	1.87
		F9UPQ9	lp_1938; Transcription regulator, LysR family	1.82
		F9USH9	lp_3060; Transcription regulator, AraC family	1.53
		F9UMJ7	lp_1020; Transcription regulator, TetR family	1.57
		F9UL40	tex; Transcription accessory protein, contains S1 RNA binding domain	−1.82
	Other regulation proteins	Q88XG5	recX; Regulatory protein RecX	2.02
		F9UM52	spx1; RNA polymerase (RNAP)-binding regulatory protein, arsenate reductase (ArsC) family, Spx subfamily	−1.59
		F9ULW6	lp_0737; Sigma 54 modulation protein/ribosomal protein S30EA	−1.53
Carbohydrate metabolism	TCA cycle	F9UMS1	fum; fumarate hydratase	−3.81
		F9UQ90	pdhD; pyruvate dehydrogenase complex, E3 component; dihydrolipoamide dehydrogenase	−1.90
		F9UQ91	pdhC; pyruvate dehydrogenase complex, E2 component; dihydrolipoamide S-acetyltransferase	−2.30
		F9UQ92	pdhB; pyruvate dehydrogenase complex, E1 component, beta subunit	−2.21
		F9UQ93	pdhA; pyruvate dehydrogenase complex, E1 component, alpha subunit	−2.12
	Pyruvate metabolism	F9UTR4	ack2; acetate kinase	−1.59
		F9UM63	pox1; pyruvate oxidase	−2.79
		P59390	ldhL2; L-lactate dehydrogenase	−3.36
		Q88VJ2	ldhD; D-lactate dehydrogenase	1.69
		F9URC8	pox3; pyruvate oxidase	−8.87
		F9UTJ5	pflB; formate C-acetyltransferase	−1.85
		P37063	pox5; pyruvate oxidase	−4.19
	Pentose phosphate pathway	F9UN42	gntK; gluconokinase	−1.99
		F9URA8	tal1; Transaldolase	−5.21
		F9UN43	lp_1251;6-phosphogluconate dehydrogenase	−7.13
		Q88S87	xfp; xylulose-5-phosphate phosphoketolase	−1.64
		F9ULK7	rbsK1; ribokinase	−1.72
	Glycolysis	Q88YY8	pgm2; phosphoglycerate mutase family protein	2.56
		F9URP6	pbg4; 6-phospho-beta-glucosidase	−1.94
		F9URP7	pbg5; 6-phospho-beta-glucosidase	−2.03
	Other	F9US84	pgmB2; Beta-phosphoglucomutase	−2.71
		F9USZ1	malS; Alpha-amylase, maltodextrins and cyclomaltodextrins	−1.61
		F9USY2	dak3; Dihydroxyacetone phosphotransferase, phosphoryl donor protein	−1.66
		F9UP85	mapA; Maltose phosphorylase	−2.73
		Q88RZ2	rbsD; D-ribose mutarotase	−1.78
		Q88S51	rhaA; L-rhamnose isomerase	−1.56
PTS system	PTS system	F9UT61	pts4ABC; PTS system trehalose-specific transporter subunit IIBC	−1.86
		F9UL45	pts9AB; PTS system, mannose-specific EIIAB component	1.73
		F9UL47	pts9D; PTS system, mannose-specific EIID component	1.82
		F9UL56	pts10A; PTS system, mannose-specific EIIA component	−1.52
		F9UL57	pts10B; PTS system, mannose-specific EIIB component	−3.91
		F9URE1	pts19D; PTS system, N-acetylglucosamine–specific EIID component	−1.79
		F9URE3	pts19B; PTS system, N-acetylglucosamine–specific EIIB component	−1.70
		F9URP8	pts20A; PTS system, cellobiose-specific EIIA component	−2.47
		F9URP9	pts20B; PTS system, cellobiose-specific EIIB component	−2.99
		F9UUH8	pts30BCA; PTS system, beta-glucoside–specific EIIBCA component	−1.71
		F9UUK9	pts35B; PTS system, galactitol-specific EIIB component	−2.50
		F9ULA5	pts35A; PTS system, galactitol-specific EIIA component	−1.54
		F9UUH6	lp_3510; PTS-associated protein	−1.80
Amino acid metabolism	Lysine biosynthesis	F9UPP5	dapE1; succinyl-diaminopimelate desuccinylase	2.86
		F9URV7	dapE2; succinyl-diaminopimelate desuccinylase	1.95
		F9UT53	cblB; cystathionine beta-lyase/cystathionine gamma-lyase	−3.54
	Sulfur amino acid metabolism	F9UQB3	iscS; Cysteine desulfurase	1.64
		F9UTH2	lp_3283; Methonine synthase (Cobalamine-independent), C-terminal domain	−2.40
		Q88UW5	gshAB; glutathione biosynthesis bifunctional protein: glutamate-cysteine ligase; glutathione synthetase	1.50
		F9UR58	oahS; O-acetylhomoserine sulfhydrylase	−2.44
	Other	Q88UT5	glyA; glycine hydroxymethyltransferase	1.53
		Q88WI3	trpD; anthranilate phosphoribosyltransferase	4.58
Nucleic acid metabolism		F9UT41	ndk; nucleoside-diphosphate kinase	−1.72
		F9UM77	gmk2; guanylate kinase	1.64
		F9URN3	lp_2762; phosphohydrolase	1.87
		F9US18	nrdD; anaerobic ribonucleoside-triphosphate reductase	2.16
		P71479	pyrR1; pyrimidine operon regulator	1.62
		F9UNA0	dgk2; Deoxynucleoside kinase	2.04
Lipid metabolism	Fatty acid biosynthesis	F9UP42	accC2; acetyl-CoA carboxylase, biotin carboxylase subunit	1.90
		Q88WG0	accD2; acetyl-CoA carboxylase, carboxyl transferase subunit beta	1.84
		F9UP44	accA2; acetyl-CoA carboxylase, carboxyl transferase subunit alpha	1.98
	Glycerol lipid metabolism	F9USY0	dak1B; dihydroxyacetone phosphotransferase, dihydroxyacetone binding subunit	−1.70
		F9USY1	dak2; dihydroxyacetone phosphotransferase, ADP-binding subunit	−1.70
		Q88ZF1	glpK; glycerol kinase	−5.69
		F9UTW9	glpF3; Glycerol uptake facilitator protein	−3.30
		Q88YD9	glpK2; Glycerol kinase 2	−3.04
		F9UT65	tagF1; CDP-glycerol glycerophosphotransferase	−2.15
		F9UT64	tagD1; glycerol-3-phosphate cytidylyltransferase	−5.85
		F9UPG2	tarL; Ribitolphosphotransferase	4.22
		F9UTW8	glpD; glycerol-3-phosphate dehydrogenase, FAD-dependent	−6.09
	Terpenoid backbone biosynthesis	Q88W46	tarI; D-ribitol-5-phosphate cytidylyltransferase	6.81
		F9URB5	dxs; 1-deoxy-D-xylulose-5-phosphate synthase	2.46
	Other	F9UT87	cfa2; Cyclopropane-fatty-acyl-phospholipid synthase	1.67
		F9UMC4	lp_0925; Acyltransferase	2.30
Two-component system		F9UMR7	citC; [citrate (pro-3S)-lyase] ligase	−1.52
		Q88XS8	citD; citrate lyase, gamma chain, acyl carrier protein	−2.43
		F9UMR9	citE; citrate lyase, beta chain	−1.82
		F9UMS0	citF; citrate lyase, alpha chain	−1.95
		Q88VM8	dltC1; D-alanine–poly(phosphoribitol) ligase subunit 2-1	−1.65
Transporter		F9USY7	mdxE; maltodextrin ABC transporter, substrate binding protein	−1.91
		F9USZ2	msmX; maltodextrin ABC transporter, ATP-binding protein	−2.23
		F9USG5	lp_3042; Multidrug ABC transporter, ATP-binding and permease protein	−1.97
		F9UM05	lp_0783; Oligopeptide ABC transporter, substrate binding protein	2.77
		F9UR50	lp_2525; ABC transporter, ATP-binding protein	1.75
		F9UPR5	lp_1945; Multidrug ABC transporter, ATP-binding protein	1.65
		F9USL7	fhuD; iron chelatin ABC transporter, substrate binding protein	1.74
		F9UMR4	citP; Citrate transport protein	−2.16
		F9UP84	malT; Carbohydrate (Maltose)/proton symporttransporter, GPH family	−2.12
Membrane protein and cell surface protein		F9URF3	lp_2663; Hypothetical membrane protein	−1.53
		F9UU92	lp_3413; Cell surface protein, CscA/DUF916 family	−1.69
		F9UTN0	lp_3359; Hypothetical membrane protein, DUF125 family	−2.24
		F9ULS6	lp_0689; Cell surface protein, lipoprotein	2.05
		F9URZ3	lp_2901; Hypothetical membrane protein	1.73
		F9UU47	lp_3360; Hypothetical membrane protein, DUF125 family	−3.35
Extracellular protein		F9USP4	lp_3134; Extracellular protein, DUF 1093 family, membrane-bound	−2.04
		F9UR45	lp_2520; Extracellular protein, NlpC/P60 family, gamma-D-glutamate-meso-diaminopimelate muropeptidase	−1.79
		F9UQA0	lp_2162; Extracellular protein, NlpC/P60 family, gamma-D-glutamate-meso-diaminopimelate muropeptidase	1.55
		F9URD4	lp_2636; Extracellular protein	1.55
		F9UUA0	lp_3421; Extracellular protein, gamma-D-glutamate-meso-diaminopimelate muropeptidase	2.39
		F9USJ3	lp_3077; Extracellular protein	2.37
		F9UQ85	lp_2145; Extracellular protein, cell wall–anchored	1.90
		F9UNC2	lp_1357; Extracellular protein, membrane-anchored	1.88
		F9UL67	zmp2; Extracellular zinc metalloproteinase, M10 family	1.96
		F9USE1	lp_3014; Extracellular transglycosylase, with LysM peptidoglycan–binding domain	1.62
		F9USH2	lp_3050; Extracellular transglycosylase, membrane-bound	3.29
Hydrolase		F9UTL5	lp_3341; Cell surface hydrolase, DUF915 family, membrane-bound	2.01
		F9UM81	gph1; Phosphohydrolase	1.58
		F9UM42	lp_0824; Hydrolase, HAD superfamily, Cof family	−3.78
		F9URL1	lp_2737; Cell surface hydrolase, DUF915 family, membrane-bound	−2.25
		F9URQ4	lp_2787; Hydrolase, HAD superfamily, Cof family	−1.96
		F9URF0	xynC; Acetyl xylosidase (Promiscuous)	1.51
		F9USG7	amd; Aminohydrolase/peptidase, M20D family	−2.33
		Q06115	cbh; Choloylglycine hydrolase	−2.05
		F9UTI3	folQ; Dihydroneopterin triphosphate pyrophosphohydrolase	−1.98
		F9UPB8	lp_1767; Glycosyl hydrolase, family 25	1.84
		F9UQI5	lp_2266; Phosphoesterase	1.75
		F9UL25	lp_0552; Phosphoesterase	−1.62
Other	Galactose metabolism and cell wall synthesis	F9UMX4	glf1; UDP-galactopyranose mutase	1.72
		F9URD9	acm2; Cell wall hydrolase/muramidase	1.51
	Inositol phosphate metabolism	Q88S38	iolG; myo-inositol 2-dehydrogenase (promiscuous)	−6.86
		Q88S37	iolE; 2-keto-myo-inositol dehydratase (promiscuous)	−2.18
		F9ULG2	lp_3608; myo-inositol 2-dehydrogenase-like (promiscuous)	−3.25
		F9ULG4	lp_3612; myo-inositol 2-dehydrogenase-like (promiscuous)	−2.95
	Amino sugar and nucleotide sugar metabolism	F9USZ6	sacK1; fructokinase	1.61
		Q88SC3	murQ1; N-acetylmuramic acid 6-phosphate etherase	−1.80
	Oxidative phosphorylation	Q88UT8	atpE; H(+)-transporting two-sector ATPase, C subunit	1.62
		F9UQR9	atpB; H(+)-transporting two-sector ATPase, A subunit	1.73
	Riboflavin metabolism	F9UNI1	ribA; 3,4-dihydroxy-2-butanone 4-phosphate synthase/GTP cyclohydrolase II	−4.71
		Q88X16	ribH; riboflavin synthase, beta chain	−4.63
	Other	F9UQZ9	lp_2463; Prophage P2b protein 18, major capsid protein	6.88
		F9UQX7	lp_2437; Prophage P2a protein 20, replication protein DnaD domain	1.95
		F9US31	lp_2953; Esterase	1.68
		F9UNU9	fthC; 5-formyltetrahydrofolate cyclo-ligase	1.98
		Q890D7	lp_0089; UPF0246 protein	1.71
		Q88V85	sepF; Cell division protein SepF	2.24
		F9UPG0	tarJ; ribitol-5-phosphate 2-dehydrogenase	10.23
		F9URL0	cah; Carbonate dehydratase	1.51
		F9URA1	rnh; Ribonuclease H	1.62
		F9UPE3	lp_1796; DegV family protein	1.50
		Q6LWH3	repB; Copy number control protein	−4.51
		Q88VB0	lp_2157; UPF0356 protein	−1.52
		F9UNZ6	tpk; Thiamin pyrophosphokinase	2.12
		F9ULU2	rsmI; Ribosomal RNA small subunit methyltransferase I	1.74
Uncharacterized protein		F9UQ40	lp_2093; Uncharacterized protein	−1.51
		F9UNU5	lp_1566; Uncharacterized protein	−1.61
		Q6LWD6	orf41; Uncharacterized protein	−1.79
		F9UPK2	lp_1872; Uncharacterized protein	−1.56
		F9UU56	lp_3372; Uncharacterized protein	−2.05
		F9UM53	lp_0837; Uncharacterized protein	−1.63
		F9UTZ1	lp_0402; Uncharacterized protein	−1.69
		F9UKY5	lp_0507; Uncharacterized protein	−1.88
		Q6LWD8	orf39; Uncharacterized protein	2.86
		Q6LWD7	orf40; Uncharacterized protein	2.66
		F9UT92	lp_3179; Uncharacterized protein	1.78
		F9URF7	lp_2667; Uncharacterized protein	1.67
		F9UQN8	lp_2333; Uncharacterized protein	1.54
		F9UN47	lp_1257; Uncharacterized protein	1.54
		F9USX1	lp_0158; Uncharacterized protein	1.51
		F9UQ59	lp_2112; Uncharacterized protein	1.66
		F9UQ60	lp_2113; Uncharacterized protein	−4.47
		F9UTE8	lp_3250; Uncharacterized protein	−3.03

^a^Category of differently expressed proteins was based on their functions annotated in the database of Uniprot and KEGG. ^b^Accession number of each protein in Uniprot database. ^c^Description of each differently expressed protein, including corresponding gene name of each protein and full protein name. ^d^FC indicates fold change of each differently expressed protein in the comparison of CCFM8610/CCFM191. Negative values indicate down-regulation of proteins, and positive values indicate up-regulation.

The proteomic dynamic changes of CCFM8610 and CCFM191 after Cd exposure were also evaluated to analyze the possible Cd tolerance mechanisms of *L*. *plantarum* strains (Tables S2 and S3). For CCFM8610, twenty-seven proteins that changed significantly (i.e., a fold change >1.5 or <−1.5, and *P* value < 0.05) by Cd stress were categorized into biological processes including global stress response, transportation, lipid, amino acid and pyrimidine metabolism and cell wall biosynthesis. For CCFM191, the abundances of 111 proteins were markedly changed after Cd exposure (i.e., a fold change >1.5 or <−1.5, and *P* value < 0.05) (Table S3). These proteins were associated with global stress response, cell wall biosynthesis and adhesion, transporters, amino acid, lipid, pyrimidine and energy metabolism, membrane proteins and extracellular proteins. It is noted that one protein with the greatest abundance change (4.45 fold up-regulation) in CCFM8610 after Cd exposure was prophage P2b protein 18, major capsid protein (lp_2463). This protein was also in higher abundance (6.88 fold) in CCFM8610 than that in CCFM191 in the untreated condition, while the abundance of this protein was not changed in CCFM191 after Cd exposure. In addition, the abundances of prophage proteins lp_0641 and lp_2444 were significantly up-regulated in CCFM191 after Cd exposure, but remained unaffected in CCFM8610. It was also observed that these two proteins were in lower abundances in CCFM8610 than CCFM191 in non-treated conditions (with fold changes of −1.87 and −3.30, respectively). This may indicate that the up-regulation of lp_0641 and lp_2444 should be considered a specific Cd response mechanism of CCFM191 itself. Such mechanism seems insufficient to protect the bacterial cell against Cd stress, as CCFM191 showed poorer Cd tolerant ability than CCFM8610.

The protein-protein interaction networks were constructed for further comparative proteomic analysis (Figs S1–S3). For the comparison between CCFM8610 and CCFM191 in natural conditions (Fig. S1), the differentially expressed proteins were categorized into 3 main clusters, including PTS system, carbohydrate metabolism and glycerol lipid metabolism. It was observed that some global stress-related proteins (including trxA1, trxA2, gshR2, msrB and msrA2) and some nucleic acid metabolism-related proteins (including pyrR1, gmk2 and ndk) were implicated in the tricarboxylic acid (TCA) sub-cluster (including fum, pdhA, pdhB, pdhC and pdhD) of carbohydrate metabolism. Since only 27 proteins changed after Cd exposure in CCFM8610, the available protein-protein interaction network is transparent (Fig. S2). The possible interaction between lp_2993 (a global stress response protein) and pdc (PadA) was observed, and the two proteins (dnaE and lp_0811) involved in pyrimidine metabolism interacted with each other. For altered protein profiles of CCFM191 after Cd exposure (Fig. S3), proteins related to glycerol lipid metabolism, pyrimidine metabolism and global stress response clustered respectively. Some differentially expressed protein profiles between CCFM8610 and CCFM191 in non-treated conditions (CCFM8610(0)/CCFM191(0); Fig. S1 and Table 2) were also altered in CCFM191 after Cd exposure (CCFM191(Cd)/CCFM191(0); Fig. S3 and Table S3), such as carbohydrate metabolism and concomitant transportation (PTS system), and a cluster of enzymes involved in glycerolipid metabolism. This might indicate that CCFM8610 exhibits an inherent resistant status to Cd even in the absence of Cd exposure, while CCFM191 displays a similar response only after Cd exposure.

### Transcription confirmation and biological phenomena

**Figure 5:**
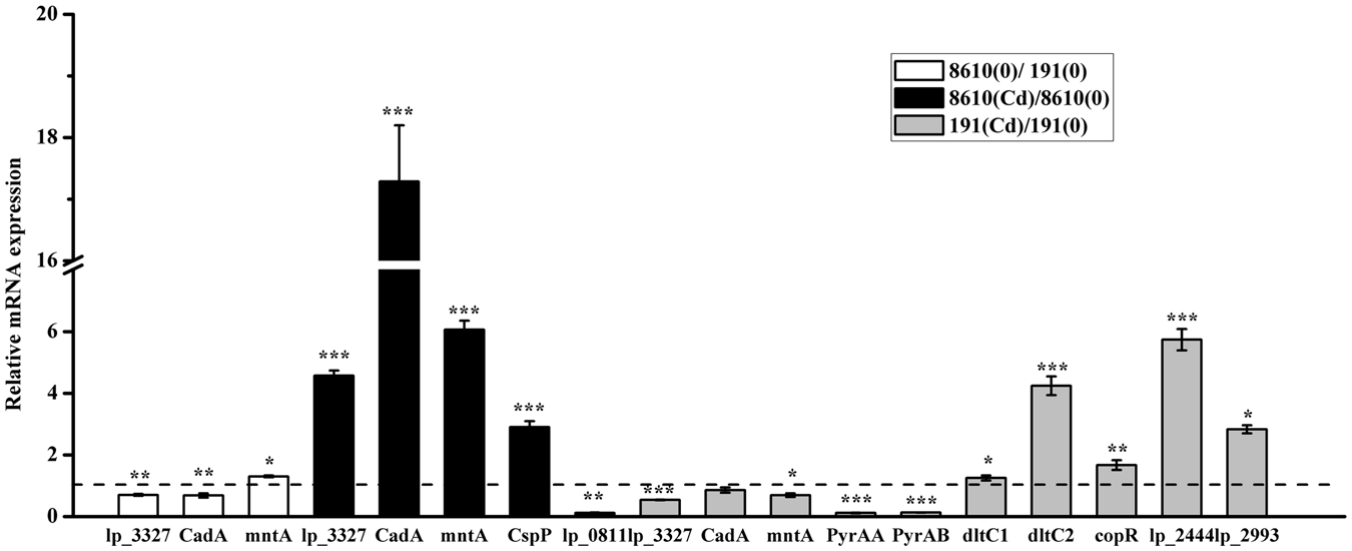
Relative mRNA expressions of genes encoding proteins altered in three comparisons, including CCFM8610(0)/CCFM191(0), CCFM8610(Cd)/CCFM8610(0) and CCFM191(Cd)/CCFM191(0). Values are expressed as mean ± SEM of 4 independent replicates. mRNA expression of denominator in each comparison is set as reference value of 1. *P < 0.05; **P < 0.01; ***P < 0.001.

Based on the genome sequence data of *L*. *plantarum* strains (https://www.ncbi.nlm.nih.gov/genome/?term=lactobacillus%20plantarum) and some well-studied Cd-tolerant microorganisms^19, 35, 36^, Cd tolerance related proteins Cd-/zinc-/cobalt-transporting ATPase (lp_3327), Cd-transporting P-type ATPase (CadA) and Cd-/manganese-transporting P-type ATPase (mntA) were further analyzed by RT-qPCR assay, as the information of these low-abundance membrane proteins is easily to be lost during proteomic analysis. Carbamoyl-phosphate synthase, pyrimidine-specific, large chain (pyrAB), Carbamoyl-phosphate synthase, pyrimidine-specific, small chain (pyrAA), D-alanine-poly (phosphoribitol) ligase subunit 2-1 (dltC1), D-alanine-poly(phosphoribitol) ligase subunit 2-2 (dltC2), Transcription regulator of CopAB ATPases (copR), Prophage P2a protein 13 (lp_2444), Nucleotide-binding protein, universal stress protein UspA family (lp_2993), DNA-directed DNA polymerase III subunit epsilon (lp_0811) and Cold shock protein 1 (CspP), were randomly selected for RT-qPCR assay to confirm the reliability of proteomic results. For this reason, the proteins in the same operon are preferred, such as pyrAA and pyrAB, and dltC1 and dltC2. The mRNA expressions of Cd tolerance-related proteins, CadA, mntA and lp_3327, showed clear up-regulation in CCFM8610 after Cd exposure, while these genes (lp_3327 and mntA) were down-regulated or remained unchanged (CadA) in CCFM191 after Cd exposure (Fig. 5). The alterations in the mRNA expressions of Csp P and lp_0811 in CCFM8610 after Cd exposure and those of PyrAA, PyrAB, dltC1, dltC2, copR, lp_2444 and lp_2993 in CCFM191 were in accordance with the corresponding changes in protein level, which verified the results of the proteomic study.

Compared with that of CCFM191, CCFM8610 showed more obvious enhancement of surface hydrophobicity after Cd exposure (Fig. S4). The autoaggregation ability of non-Cd-treated CCFM8610 was significantly greater than that of CCFM191 (*P* < 0.05, Fig. S5). Cd stress increased the autoaggregation ability of the former strain, but exhibited no marked effects on the latter. The scanning electron microscope (SEM) micrographs further confirmed these results (Fig. S6). The Cd binding ability of CCFM8610 was more than 2-fold higher than that of CCFM191 (Fig. S7). After Cd stress, the greatest amount of Cd accumulated on the external surface of the cell wall (59.22% ± 7.94% for CCFM8610 and 45.90% ± 2.03% for CCFM191, respectively) and in the space between the cell wall and the plasma membrane (12.01% ± 2.23% for CCFM8610 and 4.41% ± 0.64% for CCFM191, respectively, Fig. S8). Moreover, there is a significantly higher amount of Cd accumulated in CCFM8610 than that in CCFM191 after Cd exposure (14 times higher in the relative amount; Table S4). The contents of the other metal ions including manganese (Mn), zinc (Zn), potassium (K), sodium (Na) and magnesium (Mg) were either unchanged or showed insignificant perturbations after Cd exposure in both strains.

We also determined the intracellular reactive oxygen species (ROS) levels of two strains before and after Cd exposure (Fig. S9). The results showed that in the non-treated conditions (without Cd exposure), the intracellular ROS level of CCFM8610 was significantly lower than that of CCFM191. The former strain could also survive Cd stress with less drastic cellular response than the latter, as CCFM8610 showed a less significant increase in ROS level after Cd exposure. Seven detected hydrophobic amino acids were significantly up-regulated in CCFM8610 and CCFM191 after Cd exposure, with an exception of proline in CCFM8610 (Fig. S10). Compared with CCFM191, CCFM8610 consumed significantly less glucose in natural conditions (Fig. S11). Meanwhile, this strain showed less significant fluctuation in glucose consumption than CCFM191 after Cd exposure.

## Discussion

**Figure 6:**
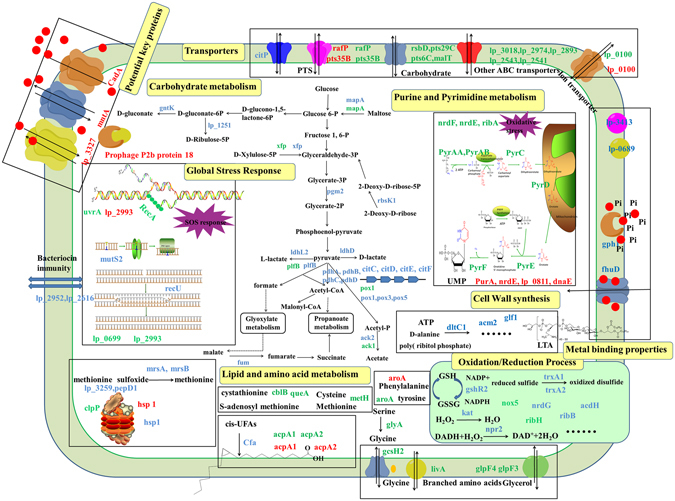
Proposed model for response mechanisms of CCFM8610 to Cd stress. Altered proteins (fold change >1.5 or <−1.5 and *P* < 0.05) in three comparisons of CCFM8610(0)/CCFM191(0), CCFM8610(Cd)/CCFM8610(0) and CCFM191(Cd)/CCFM191(0) are presented in the color of blue, red and green, respectively. Red dots represent Cd ions.

In order to explore the underlying mechanism of the intra-species differences in Cd tolerance of *L*. *plantarum* strains, we examined the proteomic profiles of CCFM8610 (strongly resistant to Cd) and CCFM191 (sensitive to Cd) in non-stimulating conditions as well as after Cd exposure using the iTRAQ approach. The results revealed that *L*. *plantarum* CCFM8610 displayed a complex biological network to tackle Cd stress, which may be related to carbohydrate, purine and pyrimidine metabolism, global stress responses, lipid and amino acid metabolism, metal binding properties, cell wall biosynthesis and transporters of the bacterial cell (Fig. 6). The Cd resistant mechanism of this strain involves a specific energy conservation survival mode, a mild induction of cellular defense and repair systems, an enhanced biosynthesis of hydrophobic amino acids, a promoted tolerance against osmotic stress and an inherent superior Cd-binding ability and effective cell wall biosynthesis ability in response to Cd stress. Potential key proteins that are important in protection of the bacterial cells against Cd toxicity were also identified.

### Carbohydrate metabolism

The comparative proteomic profiles between the two strains in the absence of Cd treatment showed that CCFM8610 has lower abundance of 24 proteins and higher abundance of only 2 proteins associated with carbohydrate metabolism (Table 2). Five enzymes (fum, pdhA, pdhB, pdhC, and pdhD) involved in TCA cycle were in lower abundance in CCFM8610. The genes that encode Pdh proteins are known to share the same operon. The repression of the pdh operon has been reported to switch TCA cycle to a branched or noncyclic anaerobic form, which is regarded as an energy conservation strategy in *E*. *coli* ^12^ and *P*. *brassicacearum* ^19^ during Cd stress. This reduces intracellular free radicals and thus protects the bacteria against Cd-induced cytotoxicity. Citrate transport protein (citP) and the four citrate lyases (citC, citD, citE and citF) were in lower abundance in CCFM8610. This corresponded to the lower citrate concentration in this strain (unpublished data). We also observed reduced abundance of 11 proteins involved in the PTS system in CCFM8610, further indicating repression of carbohydrate metabolism even in the absence of Cd stress.

The differentially expressed proteomic profiles in the two strains after Cd exposure also confirmed this self-protection mechanism of CCFM8610. No significant changes in the proteins involved in carbohydrate metabolism were found in CCFM8610 after Cd stress (Table S2), indicating that the energy conservation survival mode of this strain is beneficial for tackling Cd toxicity. In contrast, carbohydrate metabolism was markedly down-regulated in CCFM191 after Cd exposure (Table S3). This is further validated by a relative “poised” status of glucose consumption of CCFM8610 both in the presence and absence of Cd stress (Fig. S11). Such energy conservation and survival can be regarded as an inherent “poised” physiological status of the strain against environmental stresses, including Cd exposure. A previous study on the resistant mechanism of *P*. *pseudoalcaligenes* KF707 and its more resistant mutant (T5) to tellurite exposure indicated that even in the absence of tellurite, T5 cells displayed a “poised” status with altered intracellular levels of glutathione and branched-chain amino acids, along with increased resistance to other toxic metals and metabolic inhibitors^37^. Such a mechanism was believed as an inherent tolerance of the mutant strain primed for tellurite exposure, which further supports our hypothesis.

### Purine and pyrimidine metabolism

Very limited effect on pyrimidine metabolism-related proteins was observed after Cd exposure in CCFM8610. PurA, an important enzyme in the de novo pathway of purine nucleotide biosynthesis, was down-regulated in CCFM8610 after Cd exposure. NrdE, lp_0811 and dnaE, three catalytic proteins involved in DNA replication, were also changed by Cd stress. In contrast, nine purine and pyrimidine metabolism-related proteins were altered during the Cd stress responses of CCFM191 (Table S3), most of which (pyrE, pyrF, pyrD, pyrAB, pyrAA, and pyrC) showed down-regulation after Cd exposure. A similar alteration was also observed in other LAB strains in response to acid and bile stress^38, 39^. The superior Cd tolerance of CCFM8610 may be in part due to its ability to maintain a steady physiological status, including purine and pyrimidine metabolism, after Cd exposure, which can be further validated in the following analysis of its global stress responses.

### Global stress responses

Induction of global stress responses is an important strategy for bacteria to endure harsh environmental conditions. The comparative proteomic profiles between native and Cd-treated CCFM8610 showed that Cd stress caused fluctuations of only two proteins that are involved in the universal stress response, lp_2993 (up-regulated) and hsp1 (down-regulated) (Table S2). The former has also been reported to play a putative role against Cd stress in *P*. *brassicacearum* ^19^.

In contrast, CCFM191 showed more marked changes with 16 significantly altered proteins related to stress response after Cd shock (Table S3). These proteins include a key protease (Clp) functioning in maintaining cytoplasmic protein quality, four universal stress related proteins (uvrA, recA, lp_0699 and lp_2993) involving in DNA repair, 10 proteins belonging to oxidoreductases. An increase in the level of Clp, a key protease that functions in maintaining quality of cytoplasmic proteins^40^, was also found after environmental stress in other microorganisms, such as *L*. *reuteri* ^41^, *L*. *acidophilus* ^42^, and *Oenococcus oeni* ^43^.

In non-stimulating conditions, CCFM191 showed higher abundance (compared to CCFM8610) of two proteins (mutS2 and recU) involved in DNA repair, four proteins (msrA, msrB, lp_3259 and pepD1) related to protein repair, two proteins (lp_2952 and lp_2616) related to bacteriocin immunity, and one protein (hsp1) involved in stress response. In addition, a marked distinction was observed between CCFM8610 and CCFM191 in 23 oxidoreductases, indicating that these two strains display different oxidative stress status even in the absence of Cd exposure.

However, not all repair or defense proteins are stress-inducible^44^. Looking at the different nucleic acid metabolism–related and global stress response-related proteomic profiles between these two strains in non-stimulating conditions, as well as in response to Cd exposure, we conclude that compared with CCFM191, CCFM8610 is able to respond to environmental stress with weaker induction of the cellular defense and repair system. The intracellular ROS level of CCFM8610 was significantly lower than that of CCFM191 both in the presence and absence of Cd stress, which further supported our conclusion (Fig. S9).

### Lipid and amino acid metabolism

In non-stimulating conditions, cyclopropane-fatty-acyl-phospholipid synthase (cfa2) was in a higher abundance in CCFM8610 than that in CCFM191. This protein is responsible for the methylation reaction that translates preexisting *cis*-UFAs to cyclopropane fatty acids^45^. As cyclopropane fatty acids have been reported to increase acid resistance in *E*. *coli* ^46^, the relatively high expression of Cfa in CCFM8610 may be a determinant for its stress tolerance.

A chorismate mutase (aroA) and two acyl carrier proteins (acpA1 and acpA2) were up-regulated in both CCFM8610 and CCFM191 after Cd exposure (Tables S2 and S3). AroA can trigger the biosynthesis of phenylalanine and tyrosine, which in turn increases the hydrophobicity of the bacterial cell surface to prevent Cd-induced protein damage^47^. Our *in vitro* assays demonstrated a significant up-regulation of hydrophobic amino acids and surface hydrophobicity in both strains (Figures S4 and S10), which further supported this hypothesis. AcpA1 and acpA2 are well-known proteins involved in fatty acid biosynthesis and protein translation^38^. The up-regulation of these two proteins may result in a change in membrane fatty acid composition, which improves the tolerance of bacteria against environmental stress^48^.

For CCFM191, significant changes in amino acid metabolism were observed after Cd exposure (Table S3). Two proteins (glyA and gcsH2) involved in glycine biosynthesis and three proteins (cblB, queA and metH) involved sulfur amino acid metabolism were markedly up-regulated. Glycine is one of osmotic protection molecules in bacteria and has been reported to play a role in the response of metal stress^37, 49^. As Cd binds preferentially to sulfur ligand^50^, the up-regulation of sulfur amino acid metabolism may be a self-detoxification mechanism of CCFM191 against Cd exposure.

### Metal binding and cell wall biosynthesis

In conditions without Cd stress, marked differences were observed in the abundance of extracellular, membrane and cell surface proteins between CCFM8610 and CCFM191 (Table 2). It has been reported that Cd could compete for the binding sites of proteins with other metals such as iron, zinc, and calcium^51^. The higher abundance of fhuD, an iron chelatin, might improve the Cd-binding capacity of CCFM8610. Another protein with higher abundance in CCFM8610, hydrolase phosphohydrolase (gph1), can release free phosphate from lipids and precipitate Cd as CdHPO_4_ or other chemical forms onto the cell surface^19, 52^. Consistent with these analyses, our *in vitro* adsorption assays in aqueous phase solution showed that CCFM8610 possesses significantly better Cd binding ability and sequesters a higher proportion of Cd in the cellular surface than CCFM191 (Figs S7 and S8). As previously reported, this may be a self-protection of *L*. *plantarum* strain to decrease the risk of Cd-induced intracellular toxicity^53^.

As the cell wall is the first line of defense against environmental stress for bacteria^27^, the enhancement of cell wall biosynthesis may be a self-protective mechanism of *L*. *plantarum* strains in response to Cd exposure. DltC1, a protein that plays a role in lipoteichoic acid (LTA) biosynthesis, showed a 2.56-fold up-regulation in CCFM8610, which was higher than that in CCFM191 (1.71-fold, Table S3). Previous study has demonstrated that LTA can bind metal ions and affect electromechanical characteristics of the cell wall in *L*. *casei* ^54^. This can also partly explain the different Cd-binding abilities of the two *L*. *plantarum* strains tested here (Fig. S7).

The observed differences in metal binding and cell wall biosynthesis properties of the two strains were further verified by a significantly higher Cd accumulation in CCFM8610 after Cd exposure compared with CCFM191 (Table S4). The intracellular concentrations of other metals were relatively unaltered after Cd exposure. This may indicate that the Cd binding process in CCFM8610 is selective, which is in correspondence with our earlier *in vivo* study which showed that essential elements such as Ca, Zn and Mg were unaltered in the tissues of mice after oral administration of CCFM8610^10^. The adsorption of elements on the cell surface have been reported to influence the hydrophobicity and autoaggregation properties, which in turn improves the stress tolerance of the bacteria^19, 39^. This is consistent with the more significant changes in the surface properties observed in CCFM8610 after Cd exposure (Figs S4, S5 and S6).

The strong Cd tolerance of CCFM8610 might therefore be partly attributed to its inherent Cd-binding ability provided by surface proteins and the effective cell wall biosynthetic ability during Cd exposure, thus blocking the entry of this toxic metal into the cell cytoplasm.

### Transporters

Cd exposure inevitably induces osmotic stress in bacterial cells, which in turn leads to further cell damage. The tight regulation of metal import is one of the most basic mechanisms of metal homeostasis^20^, and ion transporters have been reported to play a role in the Cd stress response in *E*. *coli* ^12^. In the present study, cobalt ABC transporter ATP-binding protein (lp_0100) was observed to be down-regulated by 1.68-fold in CCFM8610 after Cd exposure, which could be regarded as a response of the strain to osmotic stress. The phosphoenolpyruvate-dependent PTS is a major carbohydrate transport system in LAB strains. Two PTS sugar transporters (rafP and pts35B) were down-regulated in CCFM8610 after Cd exposure, indicating the energy-conservation survival mode of this strain during Cd stress.

Compared with CCFM8610, CCFM191 showed extra changes in three carbohydrate transporters (pts29C, pts6C and malT), five ABC transporters (lp_3018, lp_2974, lp_2893, lp_2543 and lp_2541), one branched-chain amino acid transporter (livA) and two glycerol uptake facilitators (glpF4 and glpF3) after Cd exposure. These changes might indicate that in order to survive the Cd stress, the sensitive strain CCFM191 needs to shut down more carbohydrate transporters, optimize ABC transporters and increase the proportion of hydrophobic amino acids.

### Potential key proteins

In the non-treated conditions, the abundance of prophage P2b protein 18 (lp_2463) in CCFM8610 was 6.88-fold higher than that in CCFM191, which was the second-highest fold change among all up-regulated proteins between the two strains. In addition, this prophage protein was observed to be the most significantly up-regulated protein (4.45-fold) in CCFM8610 after Cd exposure. In the Cd-sensitive strain CCFM191, prophage P2b protein 18 remained unaffected (Table S3). In pathogenic bacteria, phage genes are related to genetic islands encoding virulence and colonization factors and play a role in the environmental adaption of the bacteria^55, 56^. Prophage-dependent thermo-resistance via plasmid integration has been reported in *Staphylococcus aureus* ^57^. The phage genes have also been observed to be up-regulated in *E*. *coli* upon Cd stress^12^. These analyses highlighted the prophage P2b protein 18 as a potential key determinant in *L*. *plantarum* strains for the response to Cd stress. The acquisition of this stress resistance-related protein by CCFM8610 is a clear benefit to the strain.

Cd-tolerance-associated proteins, including CadA, mntA and lp_3327, are believed to be Cd transporters in the cell membranes of *L*. *plantarum* strains and some well-studied Cd-tolerant microorganisms^25, 58^. Since the information of these membrane proteins is easily to be lost during proteomic analysis due to their low abundance and the insufficient extraction, the expressions of their genes were quantified by RT-qPCR (Fig. 5). The expression of CadA, a P-type ATPase which catalyzes the active efflux of Cd^2+^, was markedly up-regulated (fold change >17) after Cd exposure in CCFM8610, while in CCFM191 it was only marginally down-regulated (0.85 fold). The Cd-resistant function of CadA is well established in *S*. *aureus*, *L*. *monocytogenes* and *L*. *lactis* ^23, 59, 60^, the significantly enhanced efflux of Cd by the up-regulation of CadA might be a detoxification strategy of CCFM8610. The expression of mntA, a Cd^2+^-Mn^2+^ shared transporter, and lp_3327, a Cd-/zinc-/cobalt-transporting ATPase, were up-regulated 6.06-fold and 4.57-fold in CCFM8610 after Cd exposure, respectively. However, the expressions of these two proteins slightly decreased in CCFM191. Besides their metal transporting ability, mntA and lp_3327 have been reported to harbor multiple transmembrane domains that bind metal. Therefore, these proteins may also prevent Cd-induced cytotoxicity by Cd sequestration. This may also explain the difference of Cd tolerance between these two strains.

## Conclusion

In this study, we investigated the mechanism of Cd stress response of *L*. *plantarum* strains using comparative and functional proteomic analysis of *L*. *plantarum* CCFM8610 (strongly resistant to Cd) and *L*. *plantarum* CCFM191 (sensitive to Cd). The proteomic profiles in non-stimulating conditions and the altered proteomic profiles after Cd stress in both strains were explored. Of the total 1415 identified proteins, 206 were differentially expressed for the comparison of natural proteomic profiles of CCFM8610 and CCFM191, 27 were differently regulated in CCFM8610 after Cd exposure, and 111 were changed in CCFM191 in response to Cd stress. Both strains showed physiological alterations in energy metabolism, purine and pyrimidine metabolism, global stress responses, lipid and amino acid metabolism, metal binding properties, cell wall biosynthesis and transporters in response to Cd exposure. The underlying mechanism of the intra-species distinctions between CCFM8610 and CCFM191 on Cd tolerance can be attributed to the following aspects. (a) CCFM8610 possesses a specific energy-conservation survival mode, which can be regarded as an inherent “poised” physiological status primed for Cd exposure. (b) CCFM8610 can cope with environmental stress with mild induction of the cellular defense and repair system, which enables the strain to survive Cd exposure without drastic physiological response. (c) CCFM8610 induces the biosynthesis of hydrophobic amino acids that enhance surface hydrophobicity of the cell and prevent Cd-induced protein damage. (d) CCFM8610 has inherent superior Cd binding ability and effective cell wall structures, which promotes Cd sequestration on the surface of the cell, preventing the uptake of this toxic metal into the cytoplasm. (e) CCFM8610 exhibits a tight regulation on ion transport to withstand the Cd-induced osmotic stress. (f) Several key proteins, including prophage P2b protein 18, CadA, mntA and lp_3327, also play a potential role in Cd tolerance in CCFM8610. This study provides an overview of the Cd stress response network (Fig. 6) of *L*. *plantarum* CCFM8610 that enables this strain to be strongly resistant to Cd.

## Experimental Procedures

Cadmium chloride (CdCl_2_) and other chemicals were purchased from Shanghai Sinopharm Chemical Reagent Company (China). Trizol reagent was obtained from Ambion Life Technologies (USA). TAKARA RR047A kits were purchased from TAKARA BIO INC (China). iTaqTM Universal SYBR® Green Supermix was purchased from Bio-Rad (USA). iTRAQ reagents were purchased from Applied Biosystems (USA).

### Bacterial Strains and Growth Conditions

Twenty *L*. *plantarum* strains, including CCFM11, CCFM166, CCFM191, CCFM198, CCFM231, CCFM232, CCFM239, CCFM240, CCFM241, CCFM308, CCFM309, CCFM405, CCFM436, CCFM578, CCFM579, CCFM595, CCFM602, CCFM605, CCFM8610, and CCFM8661, were all obtained from the Culture Collections of Food Microbiology, Jiangnan University (Wuxi, China). All strains were cultured in de Man, Rogosa and Sharpe (MRS) broth (Hopebio Company, Qingdao, China) at 37 °C for 18 h routinely.

### Cd tolerance assay

As it is normally to incubate the bacteria in liquid medium for proteomic analysis^33, 61^, twenty *L*. *plantarum* strains were inoculated (2% v/v) into MRS broth containing Cd^2+^ ranging from 0 mg/L to 50 mg/L and then incubated at 37 °C. The OD_600_ values were measured at different time points until the stationary phase of the strains. The relative growth rate of each strain was expressed as a percentage of that of the control culture (without Cd exposure) which was assigned a value of 100%^33, 61^. The MIC value of each strain was determined as the lowest Cd concentration that completely inhibited the growth rate of the strains.

### Proteomics analysis

#### Whole cell protein extraction

Based on the data of Cd tolerance assay, two strains with the widest difference in Cd tolerance values were selected for proteomics analysis. The strains were grown in the Cd containing MRS broth at 37 °C and the Cd concentration was set as the 1/2 MIC value of the sensitive strain (5 mg/L) as that was established previously^33, 34^. The strains were also incubated in Cd-free media as a control.

At the early stationary phase (OD_600_ = 6.0)^38, 61^, the cells were harvested and washed twice with ice-cooled phosphate-buffered saline (PBS) solution and pelleted. The collected pellets were immediately stored at −80 °C and used for protein extraction. The cell protein was extracted as previously described with minor modifications^27, 62^. The detailed procedure of protein extraction could be found in supplementary materials.

#### Protein sample preparation and iTRAQ labeling

The detailed description of protein sample preparation and iTRAQ labeling can be seen in the supplementary materials. The workflow of the iTRAQ experiment with 9 replicates (3 biological replicates × 3 mechanical replicates in mass spectrometry runs) in each of four conditions is shown in Fig. 1.

#### Liquid chromatography tandem mass spectrometry (LC/LC−MS/MS) Analysis

The detailed procedures can be seen in the supplementary materials.

#### Protein identification and screening of differently expressed proteins

The raw data obtained from LC/LC−MS/MS analysis were processed using Thermo Proteome Discoverer software (v1.0 build 43, Thermo Fisher Scientific) and searched with Mascot (Matrix Science, London, UK) at the in-house server to perform database comparisons against *L*. *plantarum* WCFS1, based on our preliminary studies revealing that there is a utmost genome similarity between our CCFM8610 and WCFS1. The function of the identified proteins was annotated using Gene Ontology (GO) analysis, and the metabolomics pathway was analyzed using the Kyoto Encyclopedia of Genes and Genomes (KEGG) database. The threshold for differentiated expressed proteins was set as *P* < 0.05 and fold change >1.5 or <−1.5.

### Quantifications of key proteins in proteomics

The total RNA of the strains was extracted using Trizol reagent (Life Technologies). Reverse transcription was performed with TAKARA RR047A kits according to the instruction manual. The alterations of mRNA expressions were evaluated as previously reported^63^. The primers listed in Table S1 were designed using Primer 5.0 software based on the genome sequence of *L*. *plantarum* WCFS1^64^. The *L*. *plantarum* 16S rRNA gene was used as an expression control with primers specific for *L*. *plantarum* strains, and each reaction was conducted in four duplicates^65^.

### Biological phenomena

Intracellular metal accumulation, Cd binding, bacterial hydrophobicity, autoaggregation, scanning electron microscope (SEM), intracellular reactive oxygen species (ROS) production, cellular components involved in Cd binding, glucose consumption and hydrophobic amino acid production.

The detailed procedures of these assays can be seen in the material supplementary materials.

### Statistical analysis

Data were expressed as the mean ± standard error of the mean (SEM) for each group. Differences between groups were analyzed using one-way analysis of variance (ANOVA), followed by the Tukey post-hoc test. A *P* value of <0.05 was considered to indicate statistical significance.

## Electronic supplementary material

Supplementary Info
