# Supplementary material for: Identification of key proteins and pathways in cadmium tolerance of Lactobacillus plantarum strains by proteomic analysis
Source: Sci Rep. 2017 Apr 26;7:1182. doi: 10.1038/s41598-017-01180-x (PMC5430759; doi:10.1038/s41598-017-01180-x)

**Title：**Identification of key proteins and pathways in cadmium tolerance of *Lactobacillus plantarum* strains by proteomic analysis

**Authors:** Qixiao Zhai ^#^, Yue Xiao ^#^, Jianxin Zhao, Fengwei Tian, Hao Zhang, Arjan Narbad, Wei Chen ^*^

**Supplementary Experimental Procedures**

**Proteomics analysis**

**Whole cell protein extraction**

The cell pellets (~10^9^ cells) were re-suspended in 1 mL lysis buffer [4% sodium deoxycholate (SDS), 65mM dithiothreitol (DTT)], and then disrupted by intermittent sonication (200 W; 10 s on, 10 s off) for 10 min. Meanwhile, repeated freeze-thaw cycles with sonication were conducted to disrupt cells to the maximum. The lysates were centrifuged (12,000*g*, 4 °C, 30 min), and the supernatants were mixed with ice-cold acetone (supernatant: acetone=1:4, v/v) to make protein precipitated for 12 h. The protein was finally dissolved with 400 μL urea lysate solution [7 M urea，2 M thiocarbamide，0.1% phenylmethylsulfonyl fluoride (PMSF)，65 mM DTT].

**Protein sample preparation and iTRAQ labeling**

The extracted protein (100 μg in each sample) of the two strains was reduced, alkylated, hydrolysis, and labeled with iTRAQ reagents according to the instruction manual (Applied Biosystems, USA). The labeled peptides were then pooled and dried using a rotary vacuum concentrator (Christ RVC 2-25, Christ, Germany). Each sample was labeled with one of eight iTRAQ reagents (“A” round: CCFM8610 without Cd exposure, labeled as 113, 114, and 115, and CCFM8610 with Cd exposure, labeled as 116, 117, and 118; “B” round: CCFM191 without Cd exposure, labeled as 113, 114, and 115, and CCFM191 with Cd exposure, labeled as 116, 117, and 118). The aliquots in equal quantity from 12 samples were mixed and labeled with 119, and served as an intermediate to interact the samples in the “A” and “B” rounds.

**Liquid chromatography tandem mass spectrometry (LC/LC−MS/MS) Analysis**

Liquid chromatography tandem mass spectrometry (LC/LC−MS/MS) analysis was referred to previous study ^1^ and modified according to practical conditions. The peptides were resuspended with loading buffer [5 mM ammonium formate containing 2% acetonitrile (ACN); pH =10] and separated by high-pH reversed-phase liquid chromatography (RPLC, Acquity Ultra Performance LC; Waters, Milford, MA). The solvent A and solvent B is 20mM ammonium formate in water (pH=10) and 20mM ammonium formate in 100% ACN (pH=10), respectively. The gradient elution was performed with 0% to 25% B (5 to 35 min) and 25% to 45% B (35 to 48 min) on a high-pH RPLC column (C18, 3.5 µm, 150 × 2.1 mm; Waters). LC-MS/MS analyses of all fractions collected were performed with a Nano Aquity UPLC system (Waters Corporation, Milford, MA) connected to a Q Exactive hybrid quadrupole-Orbitrap mass spectrometer (Thermo Fisher Scientific, San Jose, CA) equipped with an online nano-electrospray ion source. An 8-μL peptide sample was loaded onto the Thermo Scientific Acclaim PepMap C18 column (100 μm × 2 cm; 3-μm particle size) with a flow rate of 10 μL/min for 3 min and then separated on the analytical column (Acclaim PepMap C18, 75 μm × 15 cm) with a linear gradient, from 5% B to 45% B in 45 min. The solvent A and solvent B used for separation is 5% ACN containing 0.1% formic acid and 95% ACN containing 0.1% formic acid, respectively. The column was re-equilibrated in the initial condition for 15 min. The column flow rate was maintained at 300 nL/min, and the column temperature was maintained at 40 °C. An electrospray voltage of 1.9 kV was used.

The Q Exactive mass spectrometer was operated in the data-dependent mode to switch automatically between MS and MS/MS acquisition. Survey full-scan MS spectra (m/z 300 to 1200) were acquired with a mass resolution of 70 K, followed by MS/MS scans with a resolution of 17.5 K.

**Biological phenomena: Intracellular metal accumulation, Cd binding, bacterial hydrophobicity, autoaggregation, scanning electron microscope (SEM), intracellular reactive oxygen species (ROS) production, cellular components involved in Cd binding, glucose consumption and hydrophobic amino acid production**

The surface hydrophobicity of the bacteria was determined according to previous study ^2^. Bacterial cells were either cultured in the presence or absence of Cd stress (5 ppm) until the early stationary phase (OD_600_ = 6.0), harvested and then resuspended in phosphate-buffered saline (PBS) solution (pH = 7.4) with an OD_600_ of 0.5 to 0.8. The cell suspension (3 mL) was added to 1 mL xylene and the mixture was vortexed for 2 min and incubated for 1 h at room temperature. The OD_600_ was measured, and the cell surface hydrophobicity was calculated as [(1 − OD_aqueous phase_)/OD_initial_] × 100%. To test the autoaggregation of *L. plantarum* strains, 2 mL of cell suspension (with OD_600_ adjusted to 1.0) was incubated at 37 °C. 1 mL of the upper suspension was gently collected every two hours and the OD_600_ was measured. Autoaggregation was expressed as [(1 − OD_upper suspension_)/ OD_initial_] × 100% ^3^.

To evaluate the intracellular metal ion concentrations of CCFM8610 (with and without Cd) and CCFM191 (with and without Cd), bacterial cells in the early stationary phase (OD_600_ = 6.0) were collected and digested in concentrated HNO_3_ using a microwave digestion system. Levels of Cd, manganese (Mn) and zinc (Zn) were determined by inductively coupled plasma mass spectrometry (PerkinElmer, NexIon-300X), and levels of potassium (K), sodium (Na) and magnesium (Mg) were determined by a flame or graphite furnace atomic absorption spectrophotometer (Spectr AAS or AA; Varian).

The Cd-binding capacity of two strains was measured as described in our previous study ^4^. CCFM8610 and CCFM191 were incubated in the absence of Cd stress for 18 h, and the biomass was collected by centrifugation at 8000 *g* for 20 min and washed twice with ultrapure water to obtain the cell pellets. A weighed amount of cell pellets was resuspended in ultrapure water containing 50 mg/L Cd as CdCl_2_ to give a final bacterial concentration of 1 g/L (dry weight). The samples were incubated for 1 h at 37 °C, the suspension was centrifuged at 8000*g* for 20 min, and the residual Cd concentration in the supernatant was measured by flame atomic absorption spectrophotometry (Spectr AA 220; Varian). The Cd-binding ability of the strains was expressed as milligrams per gram of bacterial cell dry weight.

The samples for scanning electron microscopy (SEM) observation were prepared as previously described ^5^. Bacterial cells were either cultured in the presence or absence of Cd stress (5 ppm) until the early stationary phase (OD_600_ = 6.0), harvested by centrifugation at 8000 *g* for 20 min, washed twice with PBS (pH = 7.2), and fixed with glutaraldehyde (2.5% v/v) for 4 h. The cells were then washed with PBS (pH = 7.2) three times and dehydrated with graded alcohols. An identical volume of isoamyl acetate was then used to displace the graded alcohols. The bacterial samples were lyophilized and a Hitachi S-3400N SEM was used to observe the cellular morphology.

The Cd binding abilities of the different cellular components of both strains were determined as our previously described with minor modifications ^5^. Bacterial cells were either cultured in the presence or absence of Cd stress (5 ppm) until the early stationary phase (OD_600_ = 6.0). Cd bound to exopolysaccharides, the external surface of the cell wall, the interior surface of the cell wall and the external surface of the cell membrane, and the interior of the protoplast was eluted separately. The Cd concentration of each cellular component was detected by inductively coupled plasma mass spectrometry (PerkinElmer, NexIon-300X) and the Cd binding ability was expressed as follows,

Cd bound by each cellular component (%)=C_1_/C_0_×100%,

Where C_0_ and C_1_ are the Cd concentrations of the intact cell and the cell component, respectively. Three independent experiments were carried out for this assay.

The intracellular reactive oxygen species (ROS) level was determined based on the incorporation of 2,7′-dichlorodihydrofluorescein diacetate (H_2_DCFDA) as previously described ^6^. Bacterial cells were either cultured in the presence or absence of Cd stress (5 ppm) until the early stationary phase (OD_600_ = 6.0). After collection, the cells were resuspended in sterile saline, and lysed by lysozyme and sonication. 150 μL of the lysate was added to a 96-well plate, followed by an addition of 10 μL H_2_DCFDA (Beyotime Biotechnology, Jiangsu, China) and sterile saline to reach a final volume of 200 μL. The reaction was performed in the dark at 25 °C for 1 h and fluorescence recorded using the Hitachi F-7000 fluorescence spectrometer. The cell number of each strain grown under Cd-treated and untreated conditions was determined by colony counting on MRS agar at 37°C for 48 h to calculate the fluorescence of 10^8^ CFU bacteria. Six biological replicates and two technical repeats were performed (12 replicates in total).

The concentration of glucose was determined by glucose assay kit (GAGO20-1KT, Sigma) according to the recommendation of the manufacturer, and the concentrations of amino acids were measured by Waters 1525-2489 High Performance Liquid Chromatography (HPLC) system. The cell density of both strains grown under Cd-treated and untreated conditions was determined by colony counting on MRS agar at 37°C for 48 h to express the concentrations of glucose and amino acids as milligrams per liter of liquid medium. Three independent experiments were carried out for both glucose and amino acids assays.

**References**

1. Qiao, J. *et al*. Quantitative iTRAQ LC–MS/MS proteomics reveals metabolic responses to biofuel ethanol in *cyanobacterial Synechocystis* sp. PCC 6803. *J Proteome Res* **11,** 5286-5300 (2012).
2. Draksler, D., Gonzáles, S., and Oliver, G. Preliminary assays for the development of a probiotic for goats. *Reprod Nutr Dev* **44,**397-405 (2004).
3. An, H. *et al*. Integrated transcriptomic and proteomic analysis of the bile stress response in a centenarian-originated probiotic *Bifidobacterium longum* BBMN68. *Mol Cell Proteomics* **13,**2558-2572 (2014).
4. Zhai, Q. *et al*. Protective effects of *Lactobacillus plantarum* CCFM8610 against acute cadmium toxicity in mice. *Appl Environ Microbiol* **79,**1508-1515 (2012).
5. Zhai, Q. et al. The cadmium binding characteristics of a lactic acid bacterium in aqueous solutions and its application for removal of cadmium from fruit and vegetable juices. RSC Adv. 6, 5990-5998 (2016).
6. Heunis, T., Deane, S., Smit, S. & Dicks, L. M. Proteomic profiling of the acid stress response in *Lactobacillus plantarum* 423. J. Proteome Res. 13, 4028-4039 (2014).

**Supplementary Tables**

Table S1 Primer sequences used for RT-qPCR^a^

| Gene name | Sequence (5′ to 3′) |
| --- | --- |
| lp_3327 | F: TAAAGAATCCGAATCACGACC  R: CTGAATGCCCAAGCTACTCC |
| CadA | F: ACTTCCGCTTAGCAATCACG  R: ATCGACGACTTCACCACCCT |
| MntA | F: TGGTTCCCATAGACGACTGA  R: GATTTAACGGAGCAACAAGC |
| PyrAB | F: CCCAAACGACGATCAGCA |
|  | R: TGACGGGAACCACTTACGC |
| PyrAA | F:GGGTTGGGATAGGCGTTATT |
|  | R: TTGGCGTAATGCTCAGTCGT |
| dltC1 | F:GCAACAATCTTAGCCGGTGTA |
|  | R:TTCAATGGGTTCCGTCCAAC |
| dltC2 | F:GGAAACGGGCACTTCAATACC |
|  | R:CATGGATGATGTTAAAGCAACG |
| copR | F:GACATAAATGACCGAAGAGGGT |
|  | R:ATAAGATGGGATGGAAGACAGC |
| lp_2444 | F:ACGCCGAAATTCTCACGAGC |
|  | R: CAGCAGCAGATTGCAGATAGGAC |
| lp_2993 | F:GTTGATGATTCTGCCGATGC |
|  | R:GCTAGGGAGCCAATAATGAGTAAGT |
| lp_0811 | F:GTTTGAGTTGCTCGGTTCCA |
|  | R:AATACGCTTGCCCACTACGG |
| Csp P | F:ACTGTAAAATGGTTCAATGCTGATA |
|  | R: TTTGGCCTTCTTCTAAGGTCTT |

^a^lp_3327, Cadmium-/zinc-/cobalt-transporting ATPase; CadA, Cadmium transporting P-type ATPase; mntA, Cadmium-/manganese-transporting P-type ATPase; pyrAB, carbamoyl-phosphate synthase, pyrimidine-specific, large chain; pyrAA, carbamoyl-phosphate synthase, pyrimidine-specific, small chain; dltC1, D-alanine–poly(phosphoribitol) ligase subunit 2-1; dltC2, D-alanine–poly(phosphoribitol) ligase subunit 2-2; copR, Transcription regulator of CopAB ATPases; lp_2444, Prophage P2a protein 13; lp_2993, Nucleotide-binding protein, universal stress protein UspA family; lp_0811, DNA-directed DNA polymerase III subunit epsilon and Csp P, Cold shock protein 1.F, forward primer; R, reverse primer.

Table S2 Differentially expressed proteins in *L. plantarum* CCFM8610 before and after Cd exposure

| Category^a^ |  | Accession^b^ | Description^c^ | FC^d^ |
| --- | --- | --- | --- | --- |
| Transcription | Transcription | P71478 | CspP; Cold shock protein 1 | 2.33 |
|  |  | F9UMI2 | cspC; Cold shock protein CspC | 2.04 |
|  |  | F9UN56 | lp_1267; Transcription regulator, MarR family | 1.75 |
| Global stress response |  | F9USV1 | hsp1; Small heat shock protein | -1.52 |
|  |  | F9USC3 | lp_2993; Nucleotide-binding protein, universal stress protein UspA family | 2.11 |
| Bacterial shape, adhesion and colonization | Bacterial adhesion | F9UP60 | lp_1697; Adherence protein, chitin-binding domain | 1.84 |
|  | Cell wall biosynthesis | Q88VM8 | dltC1; D-alanine–poly(phosphoribitol) ligase subunit 2-1 | 2.56 |
| Transporter | Carbohydrate transporter | F9UUF2 | rafP; PTS-regulated carbohydrate transporter, GPH family, raffinose/melibiose/galactose (Can switch between symport (H+) and antiport (Lactose)) | -2.03 |
|  |  | F9UUK9 | pts35B; PTS system, galactitol-specific EIIB component | -1.56 |
|  | Ion transporter | Q890D1 | lp_0100; cobalt ABC transporter ATP-binding protein | -1.68 |
|  | Other | F9URV2 | lp_2850; ABC transporter, permease protein | 1.59 |
| Amino acid metabolism | Hydrophobic amino acid metabolism | F9UMQ1 | aroA; phospho-2-dehydro-3-deoxyheptonate aldolase / chorismate mutase | 1.59 |
| Lipid metabolism | Lipid metabolism | F9UP02 | acpA1; Acyl carrier protein | 1.84 |
|  |  | F9UP36 | acpA2; Acyl carrier protein | 1.66 |
| Pyrimidine metabolism | Pyrimidine metabolism | F9ULT0 | nrdE; ribonucleoside-diphosphate reductase subunit alpha | 1.58 |
|  |  | F9UM30 | lp_0811; DNA-directed DNA polymerase III subunit epsilon | -2.41 |
|  |  | F9UPM5 | dnaE; DNA-directed DNA polymerase III subunit alpha | 1.60 |
| Other | Purine metabolism | Q88SV6 | purA; Adenylosuccinate synthetase | -1.62 |
|  | Hydrolase | F9URL1 | lp_2737; Cell surface hydrolase, DUF915 family, membrane-bound | -1.78 |
|  | Other | F9UM59 | licD; Lipooligosaccharide cholinephosphotransferase | -1.50 |
|  |  | F9UNG1 | arcT; Aminotransferase (PLP-dependent) | -1.60 |
|  |  | F9ULL2 | padA; Phenolic acid decarboxylase | 1.57 |
|  |  | F9UQZ9 | lp_2463; Prophage P2b protein 18, major capsid protein | 4.45 |
|  |  | Q88WD3 | lp_1708; UPF0337 protein | 1.55 |
|  |  | F9URN6 | lp_2765; Uncharacterized protein | 1.53 |
|  |  | F9UMT0 | lp_1123; Uncharacterized protein | 1.60 |
|  |  | F9UPQ0 | lp_1929; Uncharacterized protein | -1.72 |

^a^Category of differently expressed proteins was based on their functions annotated in the database of Uniprot and KEGG.

^b^Accession number of each protein in Uniprot database.

^c^Description of each differently expressed protein, including corresponding gene name of each protein and full protein name.

^d^FC indicates fold change of each differently expressed protein. Negative values indicate down-regulation of proteins, and positive values indicate up-regulation.

Table S3 Differentially expressed proteins in *L. plantarum* CCFM191 before and after Cd exposure.

| Category^a^ |  | Accession^b^ | Description^c^ | FC^d^ |
| --- | --- | --- | --- | --- |
| Global stress response | Degradation of misfolded proteins | Q88YH9 | clpP; ATP-dependent Clp protease proteolytic subunit | 1.51 |
|  | DNA repair | Q88YP9 | lp_0699; Nucleoid-associated protein | 1.52 |
|  |  | F9USC3 | lp_2993; Nucleotide-binding protein, universal stress protein UspA family | 3.09 |
|  |  | Q88UZ4 | recA; Protein RecA | 1.85 |
|  |  | Q88YI7 | uvrA; UvrABC system protein A | 2.12 |
|  | Oxidoreductase | F9UT43 | lp_0244; NADPH-dependent FMN reductase family protein | 2 |
|  |  | F9USL0 | lp_3096; Short-chain dehydrogenase/oxidoreductase | 1.5 |
|  |  | F9UQD7 | lp_2212; NADH-flavin reductase | 2.03 |
|  |  | F9UPR0 | lp_1939; Oxidoreductase, medium-chain dehydrogenases/reductase (MDR)/ zinc-dependent alcohol dehydrogenase-like family | 1.83 |
|  |  | F9UM47 | lp_0829; Nitroreductase family protein | 1.67 |
|  |  | F9USX2 | lp_0159; Short-chain dehydrogenase/oxidoreductase | 1.6 |
|  |  | F9URK7 | lp_2733; NADPH-dependent FMN reductase family protein | -1.5 |
|  |  | F9UMB3 | lp_0912; Enoyl-[acyl-carrier protein] reductase (NADH) | -2.09 |
|  |  | F9UU82 | lp_3403; 2,5 diketo-D-gluconic acid-like reductase, NADP–dependent (Promiscuous) | 1.5 |
|  |  | F9UUC2 | nox5; NADH oxidase | -1.7 |
|  | Other | F9UL15 | lp_0541; Ribosome-associated heat shock protein | 1.55 |
| Transcription |  | P71478 | CspP; Cold shock protein 1 | 1.65 |
|  |  | F9UU50 | copR; Transcription regulator of CopAB ATPases | 1.91 |
|  |  | F9USA1 | lp_2967; Transcription regulator, MarR family | 1.51 |
|  |  | F9ULA6 | lp_3549; Transcription regulator, DeoR family | -1.5 |
| Translation |  | Q88YX4 | rpmG; 50S ribosomal protein L33 | 1.72 |
|  |  | Q890J8 | rplI; 50S ribosomal protein L9 | 1.57 |
| DNA replication, metabolism, regulation |  | F9UQP2 | lp_2337; ATPase, AAA family | 1.58 |
|  |  | F9ULA4 | lp_0641; Prophage P1 protein 18, DNA single-strand annealing protein RecT | 4.81 |
|  |  | F9UQY3 | lp_2444; Prophage P2a protein 13 | 4.47 |
|  |  | F9URZ6 | endA; DNA-entry nuclease | 1.92 |
|  |  | F9UQA3 | recD; ATP-dependent RecD-like DNA helicase | -1.63 |
|  |  | F9USV9 | lp_0138; Nucleoside 2-deoxyribosyltransferase | 1.77 |
| Bacterial shape, adhesion, colonization and cell wall biosynthesis |  | F9USE5 | lp_3018; ABC transporter, substrate binding protein | 1.59 |
|  |  | F9UP60 | lp_1697; Adherence protein, chitin-binding domain | 2 |
|  |  | F9URD9 | acm2; Cell wall hydrolase/muramidase | 1.54 |
|  |  | Q88VM8 | dltC1; D-alanine–poly(phosphoribitol) ligase subunit 2-1 | 1.71 |
|  |  | Q88X40 | dltC2; D-alanine–poly(phosphoribitol) ligase subunit 2-2 | 1.73 |
|  |  | F9UMX4 | glf1; UDP-galactopyranose mutase | -2.31 |
| Transporter | Ion transporter | Q890D1 | lp_0100; (cobalt/Putative) ABC transporter ATP-binding protein | -2.65 |
|  | Carbohydrate transporter | F9UUF2 | RafP; PTS-regulated carbohydrate transporter, GPH family, raffinose/melibiose/galactose (Can switch between symport (H+) and antiport (Lactose)) | -1.53 |
|  |  | F9UP84 | malT; Carbohydrate (Maltose)/proton symport transporter, GPH family | 1.66 |
|  |  | F9UTP2 | pts6C; PTS system, cellobiose-specific EIIC component | -1.51 |
|  |  | F9UUH3 | pts29C; PTS system, cellobiose-specific EIIC component | -1.5 |
|  |  | F9UUK9 | pts35B; PTS system, galactitol-specific EIIB component | -1.86 |
|  | Biotin and branched-chain amino acid transporter | F9UTT8 | lp_0336; biotin transporter | -1.71 |
|  |  | F9USB7 | livA; branched-chain amino acid ABC transporter, substrate binding protein | 2.48 |
|  | Glycerol transporter | F9UMX3 | glpF4; Glycerol uptake facilitator protein | -1.64 |
|  |  | F9UTW9 | glpF3; Glycerol uptake facilitator protein | -1.63 |
|  | Other ABC transporter | F9USE5 | lp_3018; ABC transporter, substrate binding protein | 1.59 |
|  |  | F9USA6 | lp_2974; ABC transporter, substrate binding protein | 1.59 |
|  |  | F9URY7 | lp_2893; ABC transporter, ATP-binding and permease protein | 1.55 |
|  |  | F9UR63 | lp_2543; ABC transporter, ATP-binding protein | 1.95 |
|  |  | F9UR61 | lp_2541; ABC transporter, substrate binding protein | 1.97 |
| Amino acid metabolism | Hydrophobic amino acid metabolism | F9UMQ1 | aroA; phospho-2-dehydro-3-deoxyheptonate aldolase / chorismate mutase | 1.74 |
|  | Sulfur amino acid metabolism | F9UT53 | cblB; cystathionine beta-lyase / cystathionine gamma-lyase | 1.92 |
|  |  | Q88V04 | queA; S-adenosylmethionine:tRNA ribosyltransferase-isomerase | 1.53 |
|  |  | F9UN77 | metH; homocysteine S-methyltransferase (cobalamin-dependent) | 1.51 |
|  | Other amino acid metabolism | Q88UT5 | glyA; glycine hydroxymethyltransferase | 1.64 |
|  |  | Q88TY1 | dsdA; Probable D-serine dehydratase | 1.63 |
|  |  | O08322 | argF; ornithine carbamoyltransferase | 1.62 |
|  |  | Q88Y39 | asnA; aspartate-ammonia ligase | 1.64 |
|  |  | F9UQQ5 | gcsH2; Glycine cleavage system, H protein | 1.68 |
|  |  | Q88Y39 | asnA; aspartate-ammonia ligase | 1.64 |
| Carbohydrate metabolism | Carbohydrate metabolism | F9UP85 | mapA; Maltose phosphorylase | 1.55 |
|  |  | F9URA8 | tal1; Transaldolase | -1.75 |
|  |  | F9USY2 | dak3; Dihydroxyacetone phosphotransferase, phosphoryl donor protein | -1.58 |
|  |  | Q88S87 | lp_3551; Probable phosphoketolase 2 | -1.64 |
|  | Pyruvate metabolism | F9UT17 | ack1; Acetate kinase | 2.05 |
|  |  | F9UTV9 | accB3; acetyl-CoA carboxylase, biotin carboxyl carrier protein | 1.6 |
|  |  | F9UM63 | pox1; pyruvate oxidase | -1.5 |
|  |  | F9UTJ5 | pflB; formate C-acetyltransferase | -1.72 |
|  |  | F9UTJ6 | pflA; Pyruvate formate-lyase-activating enzyme | -2 |
|  | Pentose phosphate pathway | F9ULK7 | rbsK1; Ribokinase | 1.54 |
|  | Other | Q88RZ2 | rbsD; D-ribose mutarotase | 1.67 |
| Lipid metabolism | CoA biosynthesis | Q88S37 | iolE; Inosose dehydratase | -1.53 |
|  |  | Q88S38 | iolG; Inositol 2-dehydrogenase/D-chiro-inositol 3-dehydrogenase | -2.2 |
|  |  | F9ULG2 | lp_3608; Myo-inositol 2-dehydrogenase-like (Promiscuous) | -1.9 |
|  |  | F9ULG4 | lp_3612; Myo-inositol 2-dehydrogenase-like (Promiscuous) | -1.73 |
|  | Acyl transfer | F9UP02 | acpA1; Acyl carrier protein | 1.84 |
|  |  | F9UP36 | acpA2; Acyl carrier protein | 2 |
|  | Glycerophospholipid metabolism | F9USY0 | dak1B; dihydroxyacetone phosphotransferase, dihydroxyacetone binding subunit | -1.79 |
|  |  | F9UQP7 | glpQ3; Glycerophosphodiester phosphodiesterase | -1.52 |
|  |  | F9UTW8 | glpD; Glycerol-3-phosphate dehydrogenase, FAD-dependent | -2.85 |
|  |  | F9USY1 | dak2; dihydroxyacetone phosphotransferase, ADP-binding subunit | -1.57 |
|  |  | Q88ZF1 | glpK; glycerol kinase 1 | -1.7 |
|  | Isoprenoid biosynthesis | Q88Z91 | ispE;4-diphosphocytidyl-2-C-methyl-D-erythritol kinase | -1.94 |
| Pyrimidine metabolism |  | P77889 | pyrE; orotate phosphoribosyltransferase | -1.57 |
|  |  | P77888 | pyrF; orotidine-5'-phosphate decarboxylase | -1.66 |
|  |  | P77887 | pyrD; Dihydroorotate dehydrogenase A (fumarate) | -1.66 |
|  |  | P77886 | pyrAB; carbamoyl-phosphate synthase, pyrimidine-specific, large chain | -2.08 |
|  |  | P77885 | pyrAA; carbamoyl-phosphate synthase, pyrimidine-specific, small chain | -2.17 |
|  |  | P77884 | pyrC; dihydroorotase | -1.97 |
|  |  | F9ULS9 | nrdF; ribonucleoside-diphosphate reductase, beta chain | 1.57 |
|  |  | F9UNI1 | ribA; GTP cyclohydrolase-2 | 1.81 |
|  |  | F9ULT0 | nrdE; ribonucleoside-diphosphate reductase subunit alpha | 1.86 |
| Membrane protein and cell surface protein | Membrane protein without specific function | F9ULM1 | lp_3678; Cell surface protein, CscA/DUF916 family | 2.02 |
|  |  | F9ULM2 | lp_3679; Cell surface protein, CscB family | 3.15 |
|  |  | F9UNG7 | lp_1417; Hypothetical membrane protein | -2.2 |
|  |  | F9URZ3 | lp_2901; Hypothetical membrane protein | 1.96 |
|  | Signal transduction | F9ULL8 | sip3; Signal peptidase I; Signal peptidase I | 1.55 |
| Extracellular protein | Extracellular protein | F9USP4 | lp_3134; Extracellular protein, DUF 1093 family, membrane-bound | 1.9 |
|  |  | F9UTC2 | lp_3218; Extracellular protein, membrane-anchored | 1.53 |
|  |  | F9UTQ8 | lp_0304; Extracellular transglycosylase | 1.81 |
| Other | Hydrolase | F9UR44 | lp_2519; Cell surface hydrolase, DUF915 family, membrane-bound | 1.77 |
|  |  | F9URQ4 | lp_2787; Hydrolase, HAD superfamily, Cof family | -1.65 |
|  | Other | F9UUK7 | lp_3545; D-arabitol-phosphate dehydrogenase | -1.96 |
|  |  | F9ULK4 | srlD2; sorbitol-6-phosphate 2-dehydrogenase | -1.75 |
|  |  | Q88X16 | ribH; 6,7-dimethyl-8-ribityllumazine synthase | 2.39 |
|  |  | F9ULL2 | padA; Phenolic acid decarboxylase | 2.71 |
|  |  | F9US31 | lp_2953; Esterase | 1.55 |
|  |  | F9UQA4 | lp_2169; Uncharacterized protein | 2.09 |
|  |  | F9UR87 | lp_2573; Uncharacterized protein | 1.56 |
|  |  | F9URN6 | lp_2765; Uncharacterized protein | 1.79 |
|  |  | F9UTE8 | lp_3250; Uncharacterized protein | -1.64 |
|  |  | Q88V12 | lp_2275; UPF0297 protein | 1.51 |

^a^Category of differently expressed proteins was based on their functions annotated in the database of Uniprot and KEGG.

^b^Accession number of each protein in Uniprot database.

^c^Description of each differently expressed protein including corresponding gene name of each protein and full protein name.

^d^FC indicates fold change of each differently expressed protein. Negative values indicate down-regulation of proteins, and positive values indicate up-regulation.

**Table S4** Relative abundance of intracellular metal ions of two *Lactobacillus plantarum* strains with or without Cd exposure

| **Metal ions** | **Relative abundance of intracellular metal ions^a^** | | |
| --- | --- | --- | --- |
|  | **8610(0)/191(0)** | **8610(Cd)/8610(0)** | **191(Cd)/191(0)** |
| Cd | 0.10±0.02* | 25777±1778* | 1826±75* |
| Mn | ND | 1.48±0.05 | ND |
| Zn | 1.04±0.06 | 0.81±0.04 | 0.63±0.03* |
| K | 0.78±0.02* | 1.00±0.02 | 1.04±0.01 |
| Na | 1.80±0.06* | 0.79±0.09 | 0.61±0.01* |
| Mg | 1.14±0.07 | 1.53±0.15* | 1.05±0.05 |

^a^The “*” represented the significant difference (*P* <0.05) for each comparison. Data are expressed as the mean ± SEM of three independent experiments per assay. “ND”, the concentration of Mn in strain CCFM191 was undetectable because its values were lower than the determination limit.

**Supplementary Figure legends**

**Figure S1 Protein-protein interaction network of differentially expressed proteins between *L. plantarum* CCFM8610 and CCFM191.** Nodes represent proteins. Edges represent protein-protein association. These associations are meant to be specific and meaningful, i.e. proteins jointly contribute to a shared function. The meanings of different line colors are as follows: **___**, from curated databases; **___**, experimentally determined; **___**, gene neighborhood; **___**, gene fusions; **___**, gene co-occurrence; **___**, text mining; **___**, co-expression; **___**, protein homology.

**Figure S2 Protein-protein interaction network of differentially expressed proteins in *L. plantarum* CCFM8610 after Cd exposure.** Nodes represent proteins. Edges represent protein-protein association. These associations are meant to be specific and meaningful, i.e. proteins jointly contribute to a shared function. The meanings of different line colors are as follows: **___**, from curated databases; **___**, experimentally determined; **___**, gene neighborhood; **___**, gene fusions; **___**, gene co-occurrence; **___**, text mining; **___**, co-expression; **___**, protein homology.

**Figure S3 Protein-protein interaction network of differentially expressed proteins in *L. plantarum* CCFM191 after Cd exposure.** Nodes represent proteins. Edges represent protein-protein association. These associations are meant to be specific and meaningful, i.e. proteins jointly contribute to a shared function. The meanings of different line colors are as follows: **___**, from curated databases; **___**, experimentally determined; **___**, gene neighborhood; **___**, gene fusions; **___**, gene co-occurrence; **___**, text mining; **___**, co-expression; **___**, protein homology.

**Figure S4 Alterations of surface hydrophobicity of *L. plantarum* CCFM8610 and CCFM191 after Cd exposure.** Values are mean ± SEM from 6 replicates (3 biological replicates × 2 technical replicates). Different letters indicate significant difference (*P* <0.05) among groups.

**Figure S5 Alterations of autoaggregation of *L. plantarum* CCFM8610 and CCFM191 after Cd exposure.** Values are mean ± SEM from 6 replicates (3 biological replicates × 2 technical replicates). Different letters indicate significant difference (*P* <0.05) among groups.

**Figure S6 Scanning electron microscopic images of *L. plantarum* CCFM8610 and CCFM191 in the absence and presence of Cd stress.** (A) CCFM8610 before Cd exposure. (B) CCFM8610 after Cd exposure. (C) CCFM191 before Cd exposure. (D) CCFM191 after Cd.

**Figure S7 Cd-binding abilities of *L. plantarum* CCFM8610 and CCFM191.** Values are mean ± SEM from 3 independent experiments. Different letters indicate significant difference (*P* <0.05) between groups.

**Figure S8 Cd-binding abilities of the cellular components of *L. plantarum* CCFM8610 and CCFM191.** Values are mean ± SEM from 3 independent experiments. Different letters indicate significant difference (*P* <0.05) between groups.

**Figure S9 Intracellular reactive oxygen species (ROS) levels of *L. plantarum* CCFM8610 and CCFM191 in the absence and presence of Cd stress.** Values are mean ± SEM from 12 replicates (6 biological replicates×2 technical replicates). Different letters indicate significant difference (*P* <0.05) among groups. Each value is expressed as percentage of that of CCFM8610 (0).

**Figure S10 Concentrations of hydrophobic amino acids of *L. plantarum* CCFM8610 and CCFM191 in the absence and presence of Cd stress.** Values are mean ± SEM from 3 independent experiments. Different letters indicate significant difference (*P* <0.05) among groups. Ala, alanine; Tyr, tyrosine; Val, valine; Phe, phenylalanine; Ile, isoleucine; Leu, leucine; Pro, proline.

**Figure S11 Glucose consumption of *L. plantarum* CCFM8610 and CCFM191 in the absence and presence of Cd stress.** Values are mean ± SEM from 3 independent experiments. Different letters indicate significant difference (*P* <0.05) among groups.

**Figures**

Figure S1


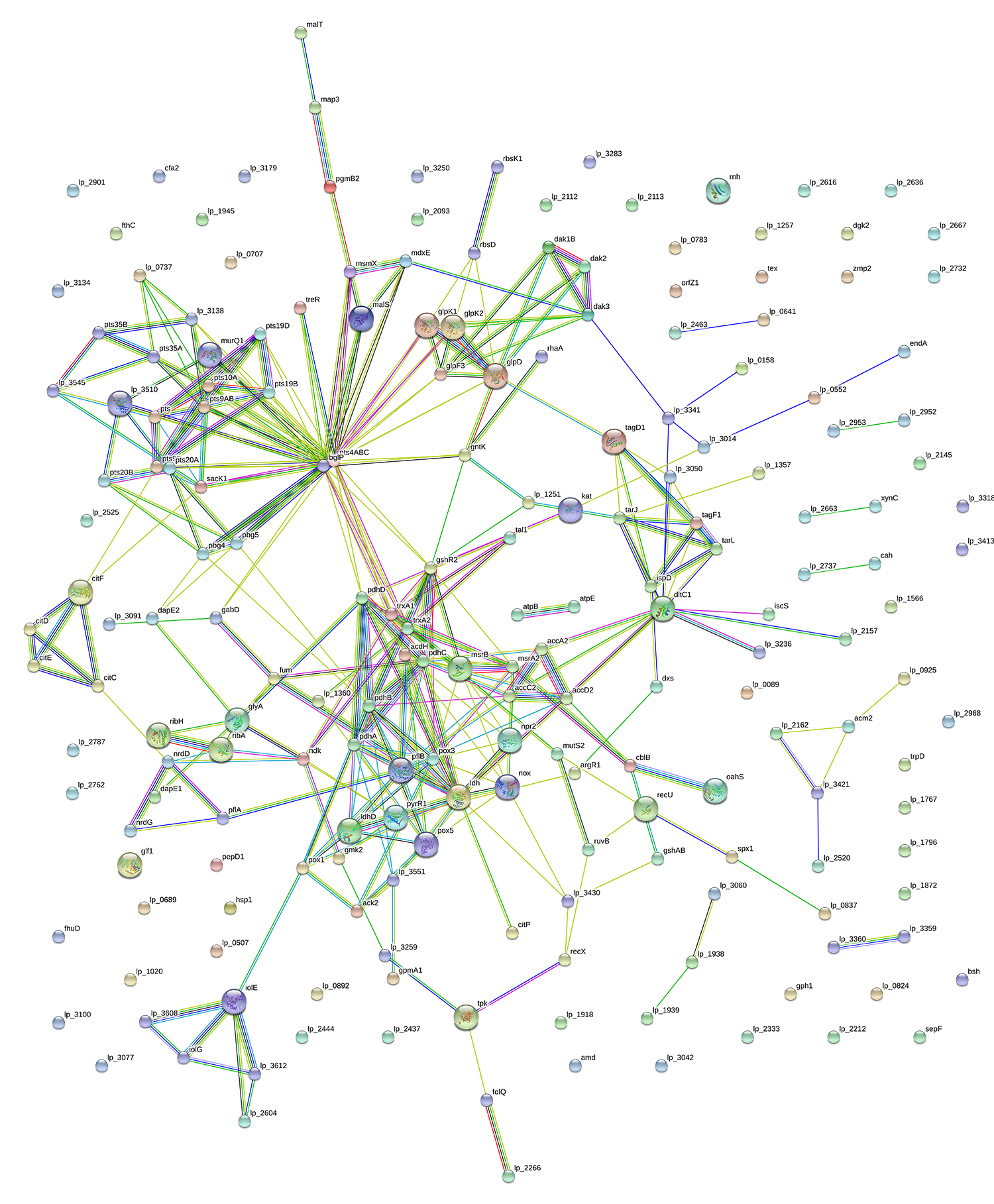


Figure S2


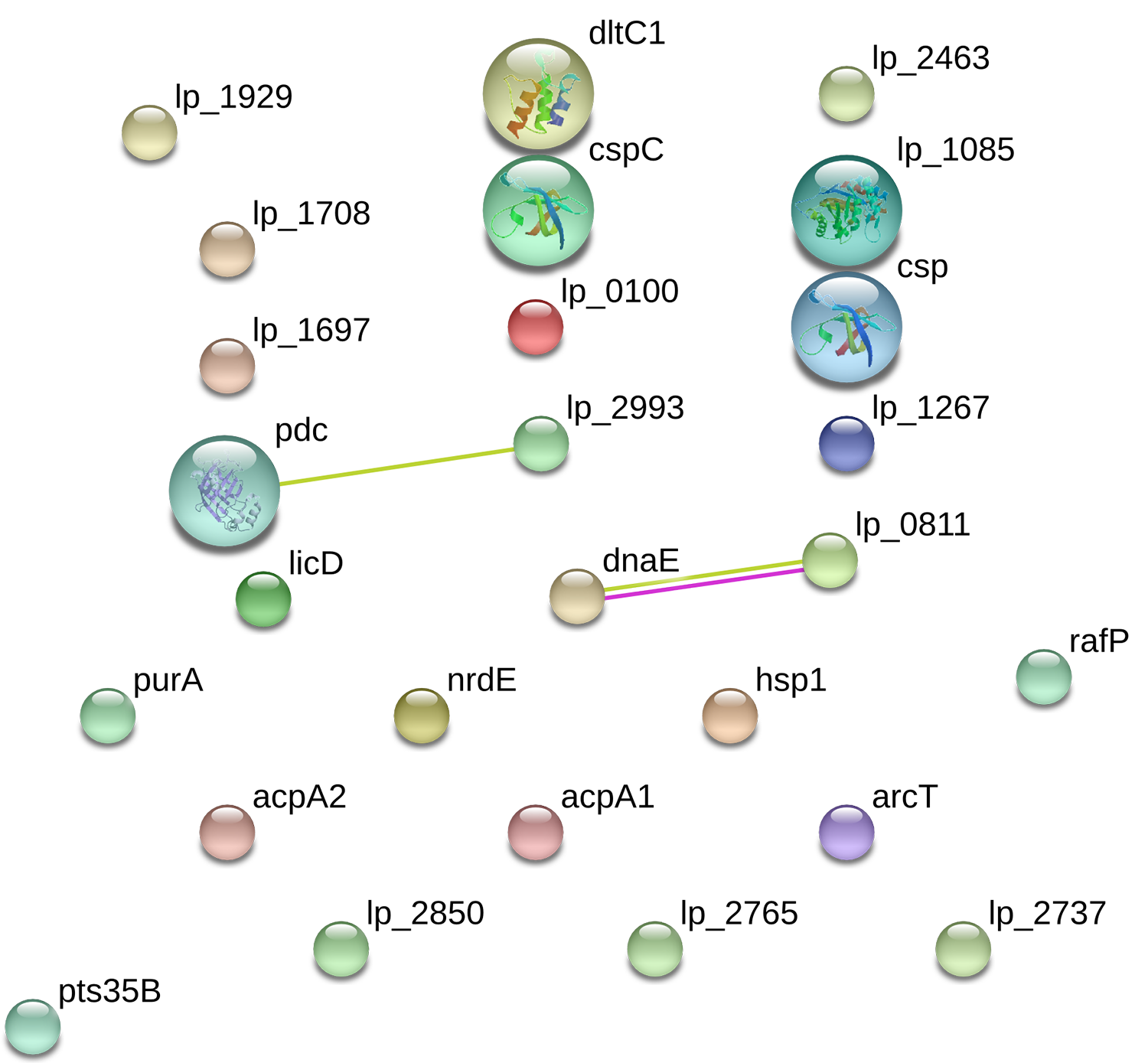


Figure S3


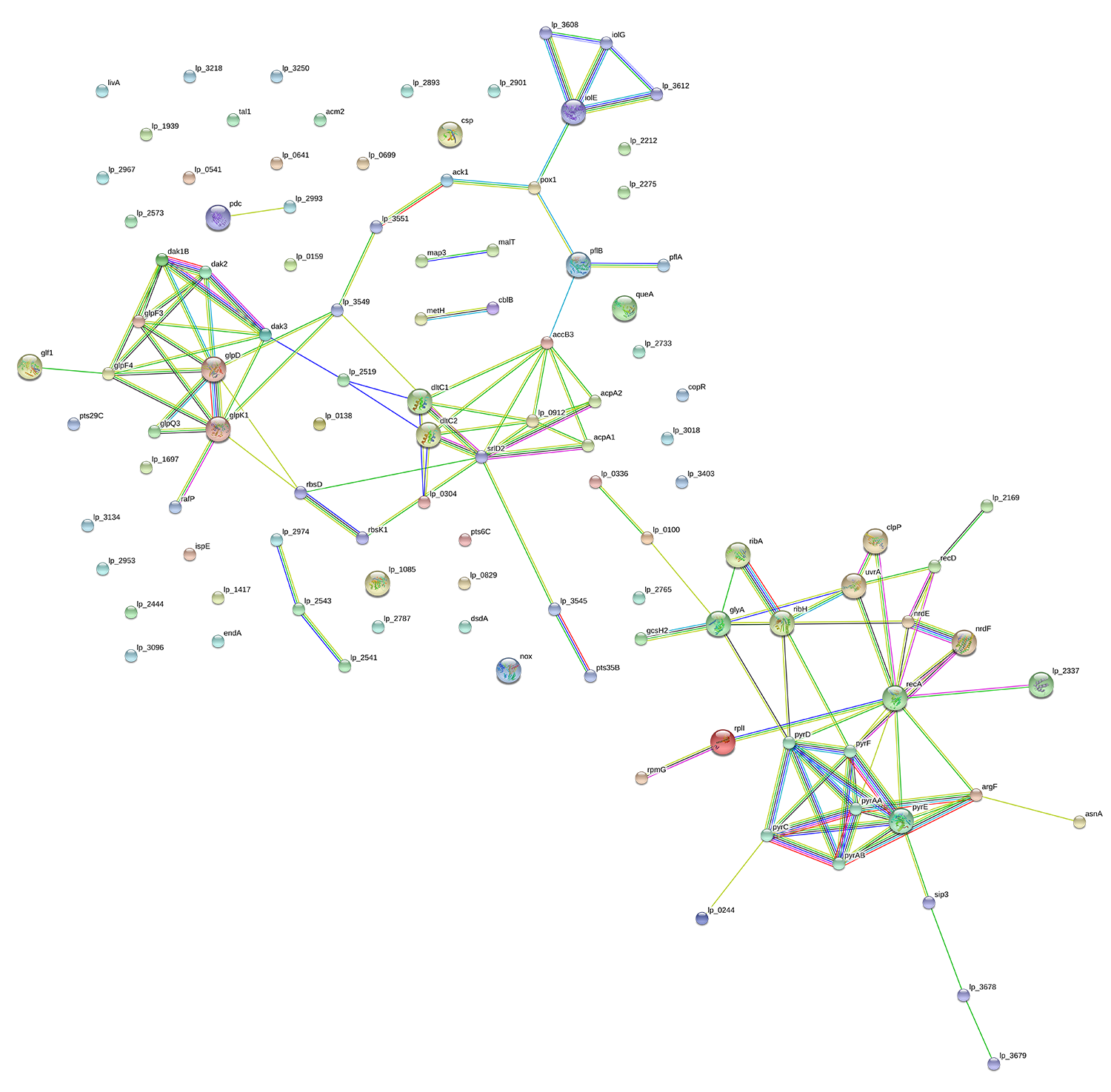


Figure S4


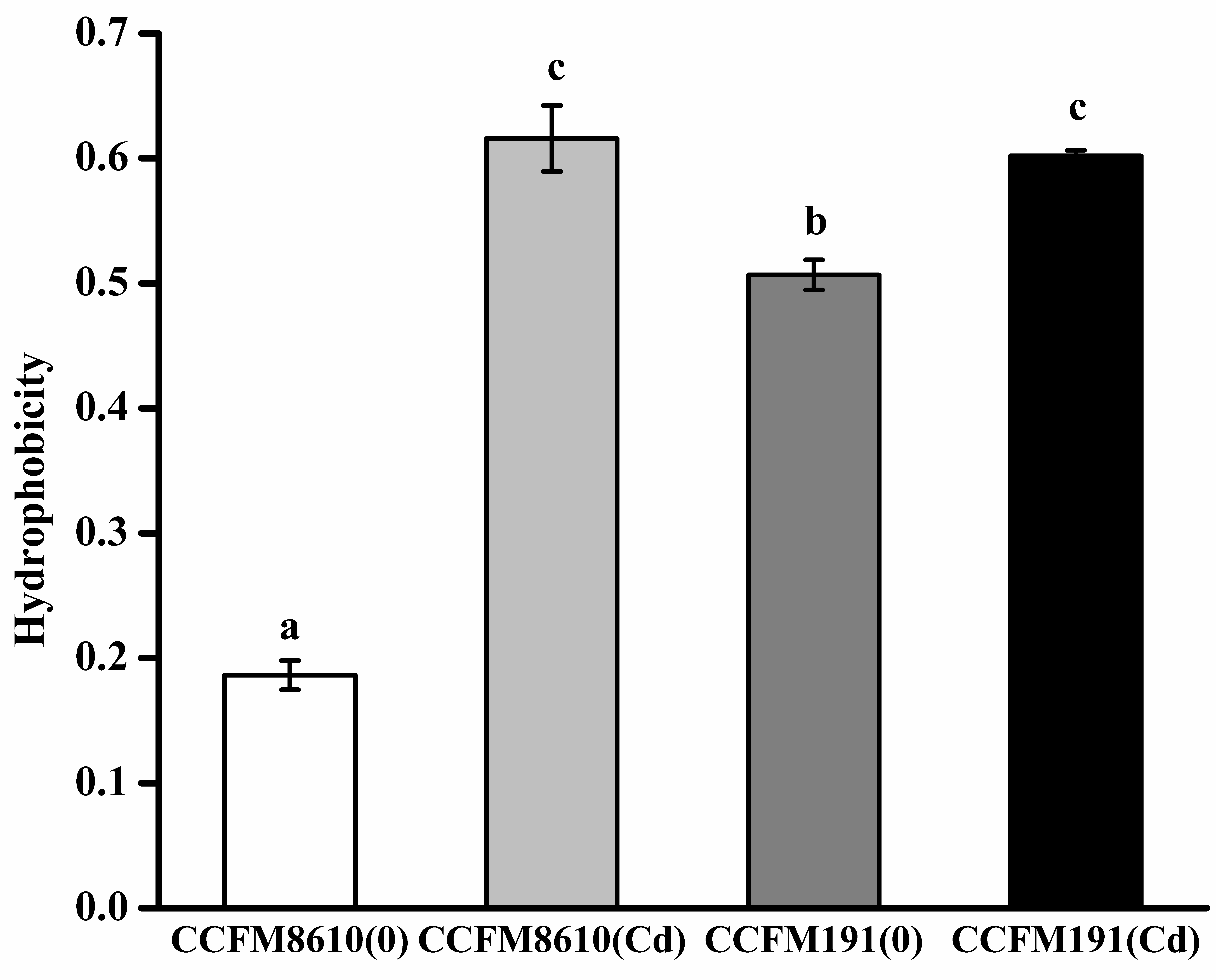


Figure S5


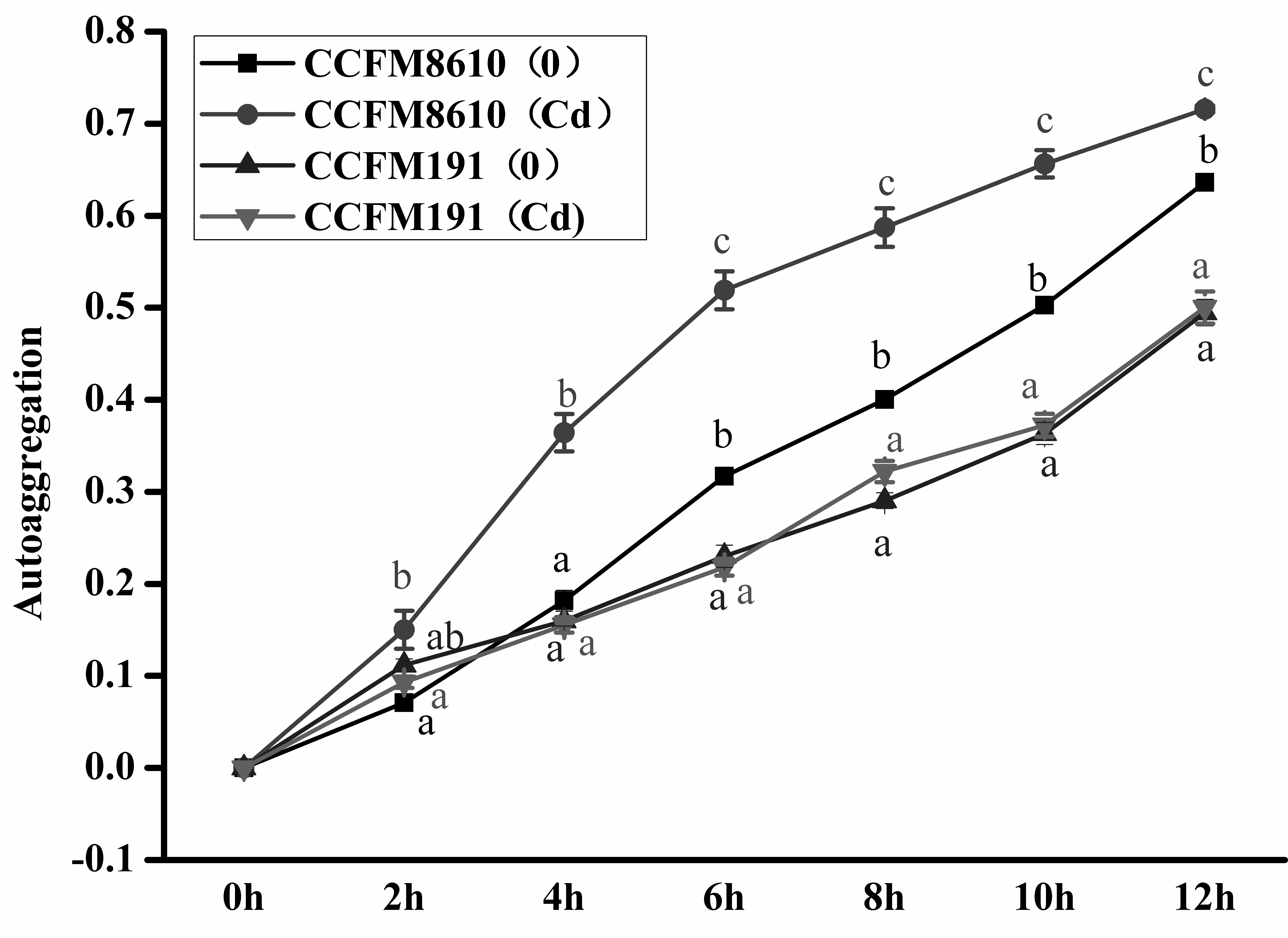


Figure S6


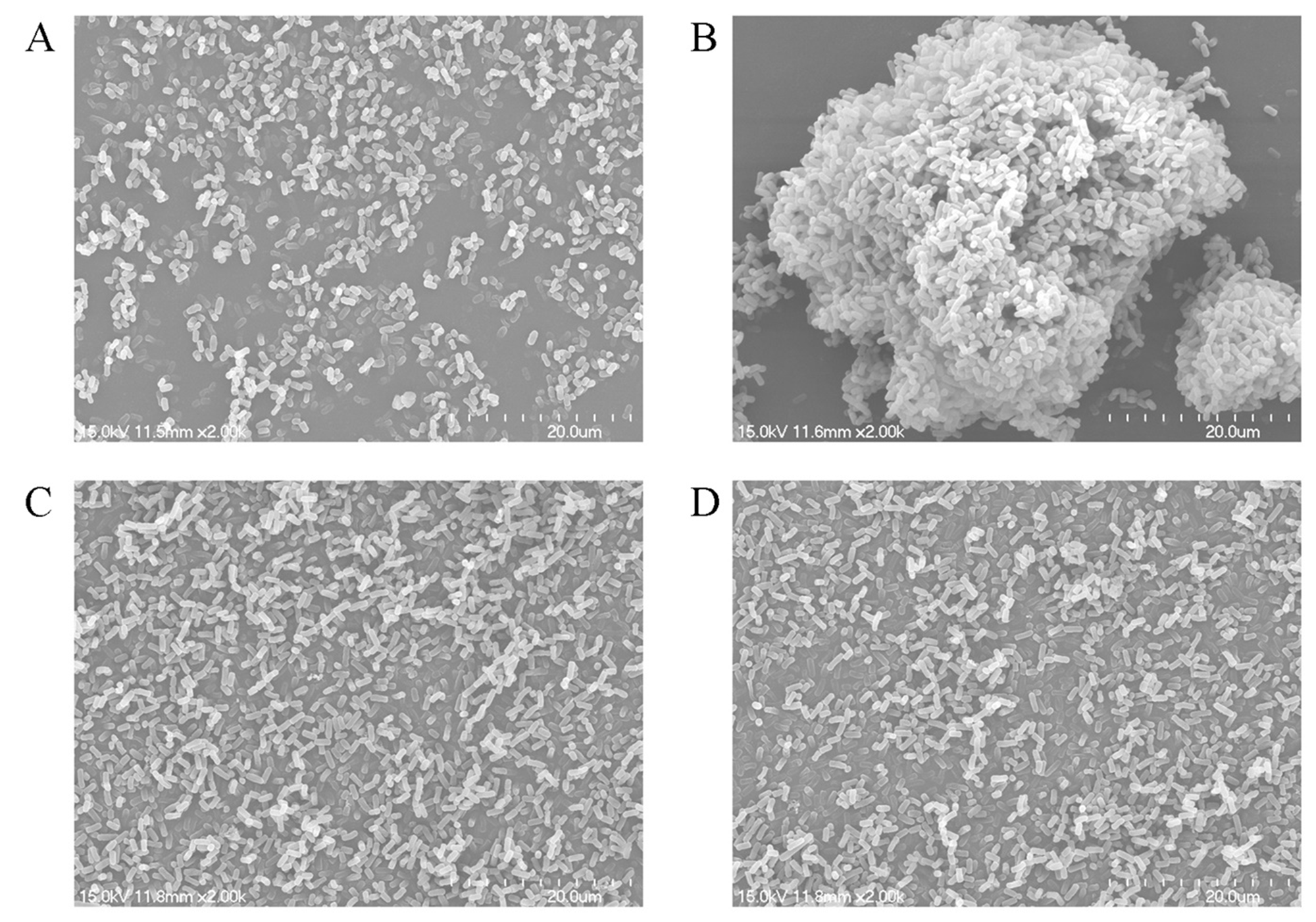


Figure S7


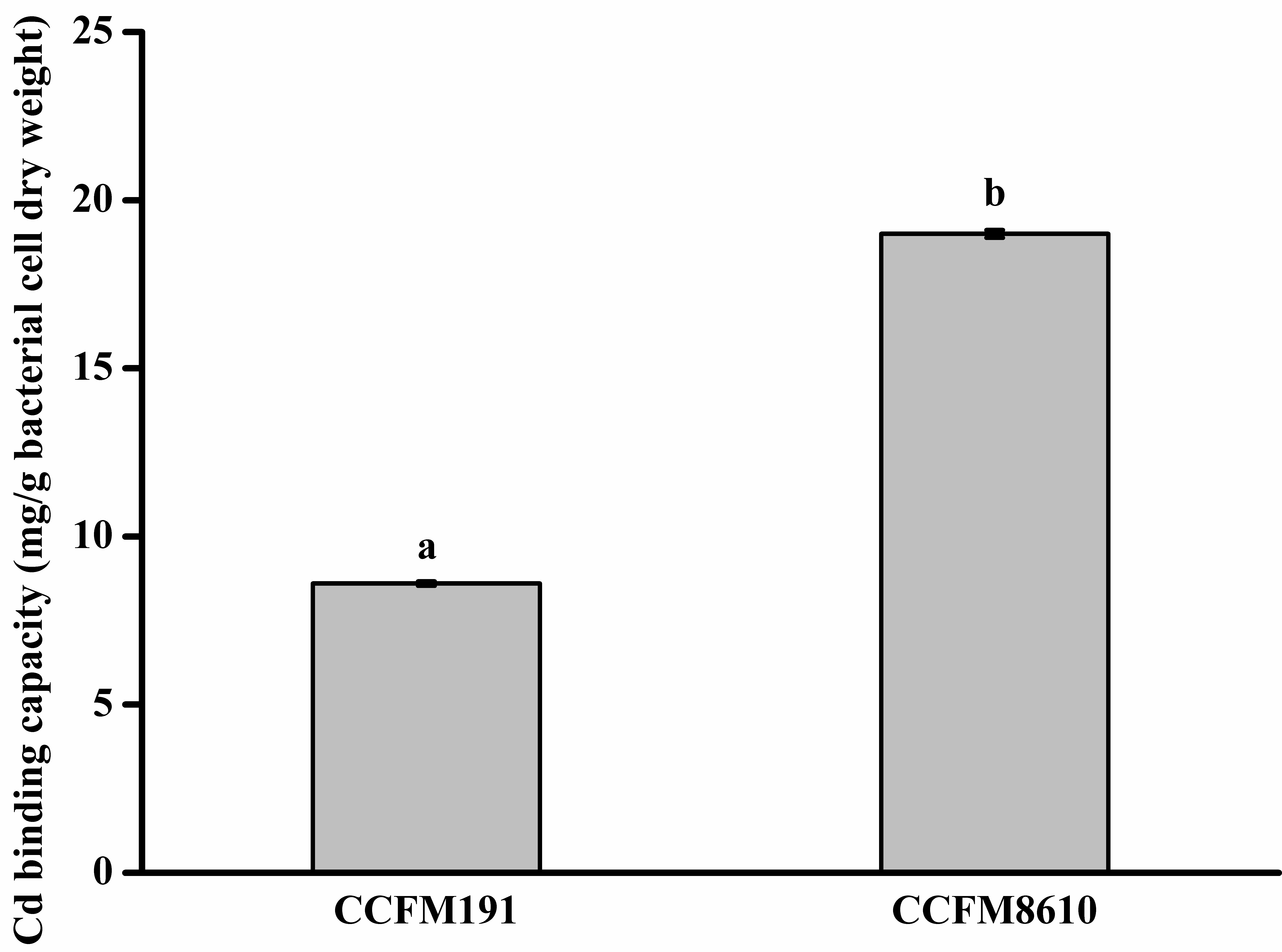


Figure S8


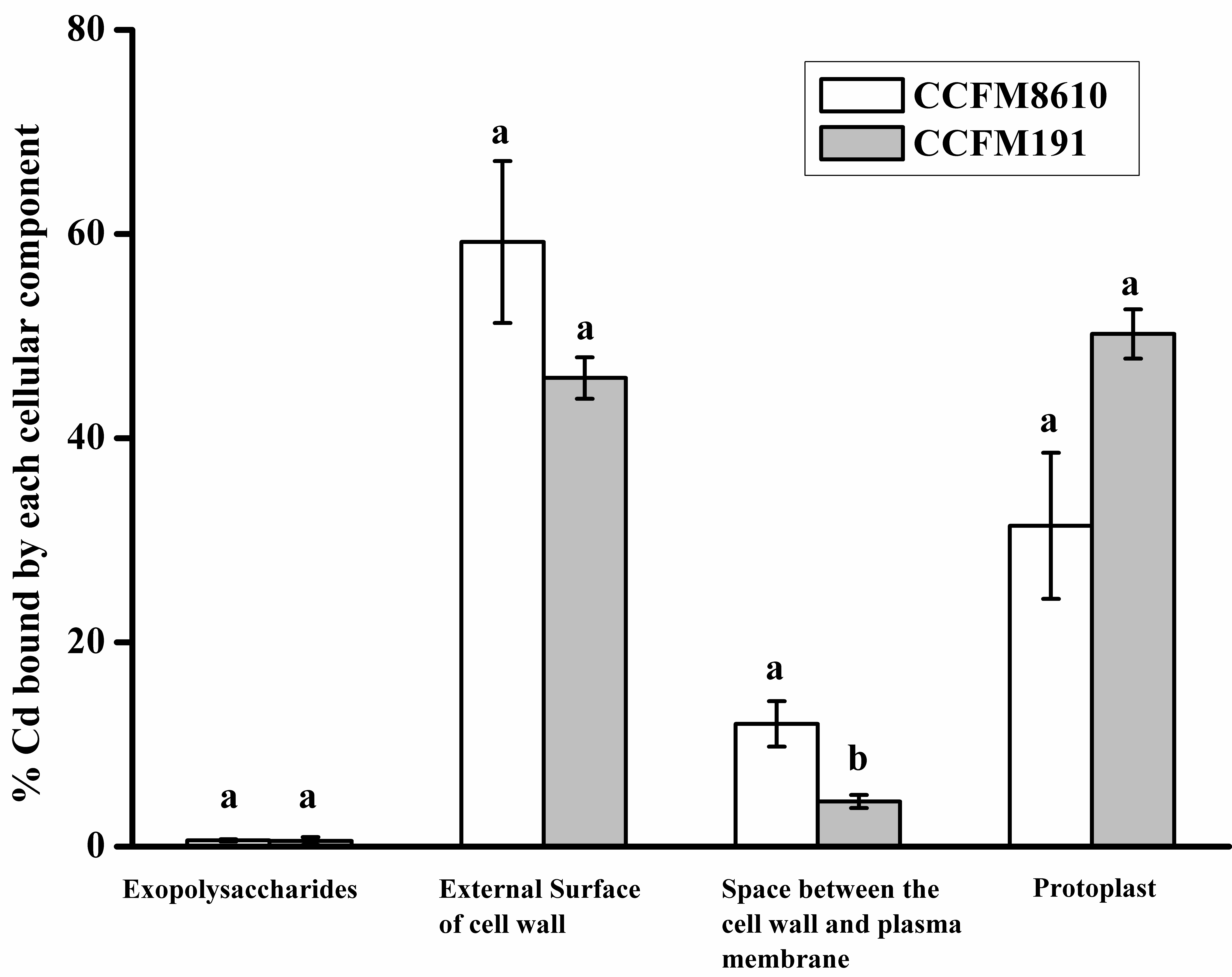


Figure S9


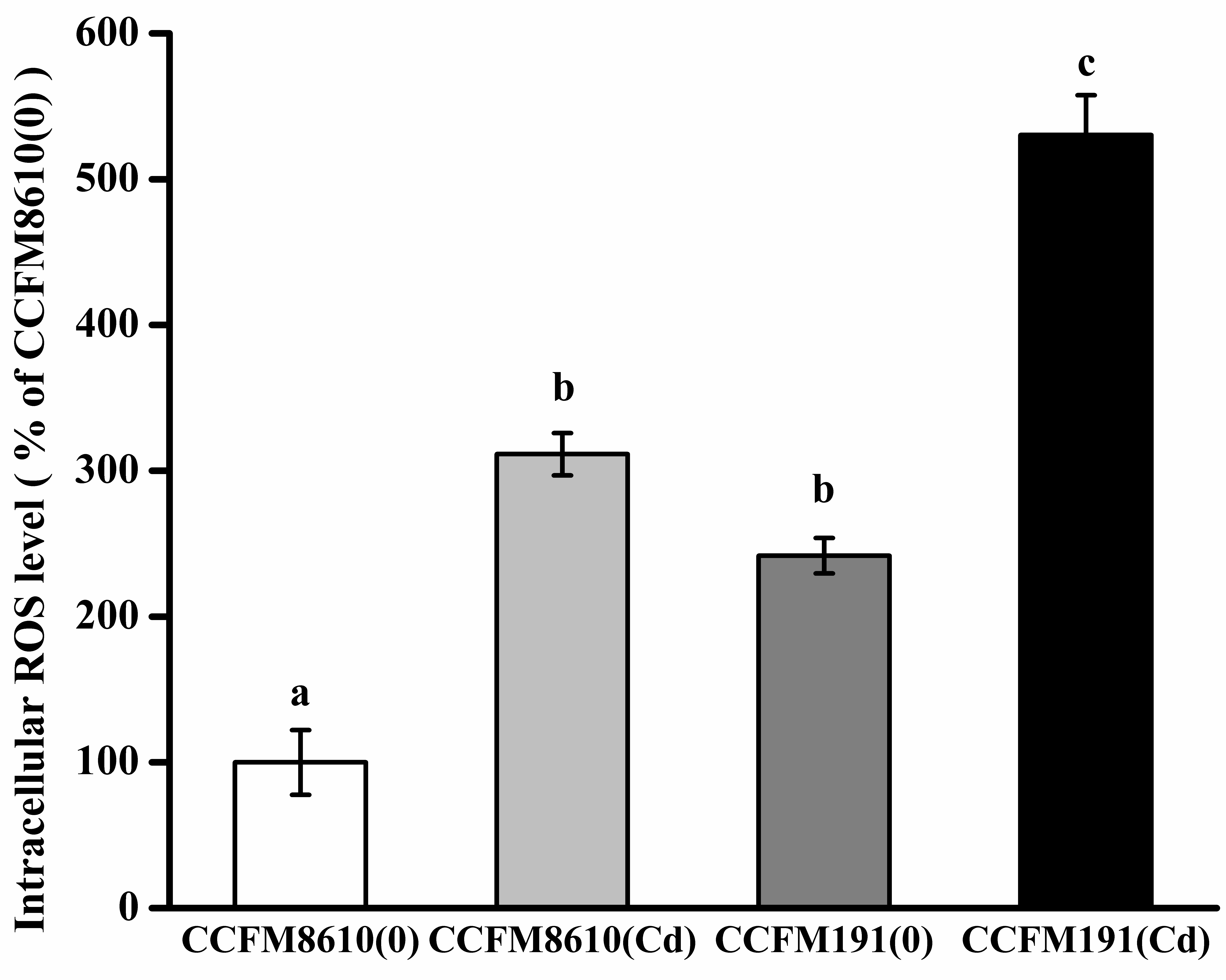


Figure S10


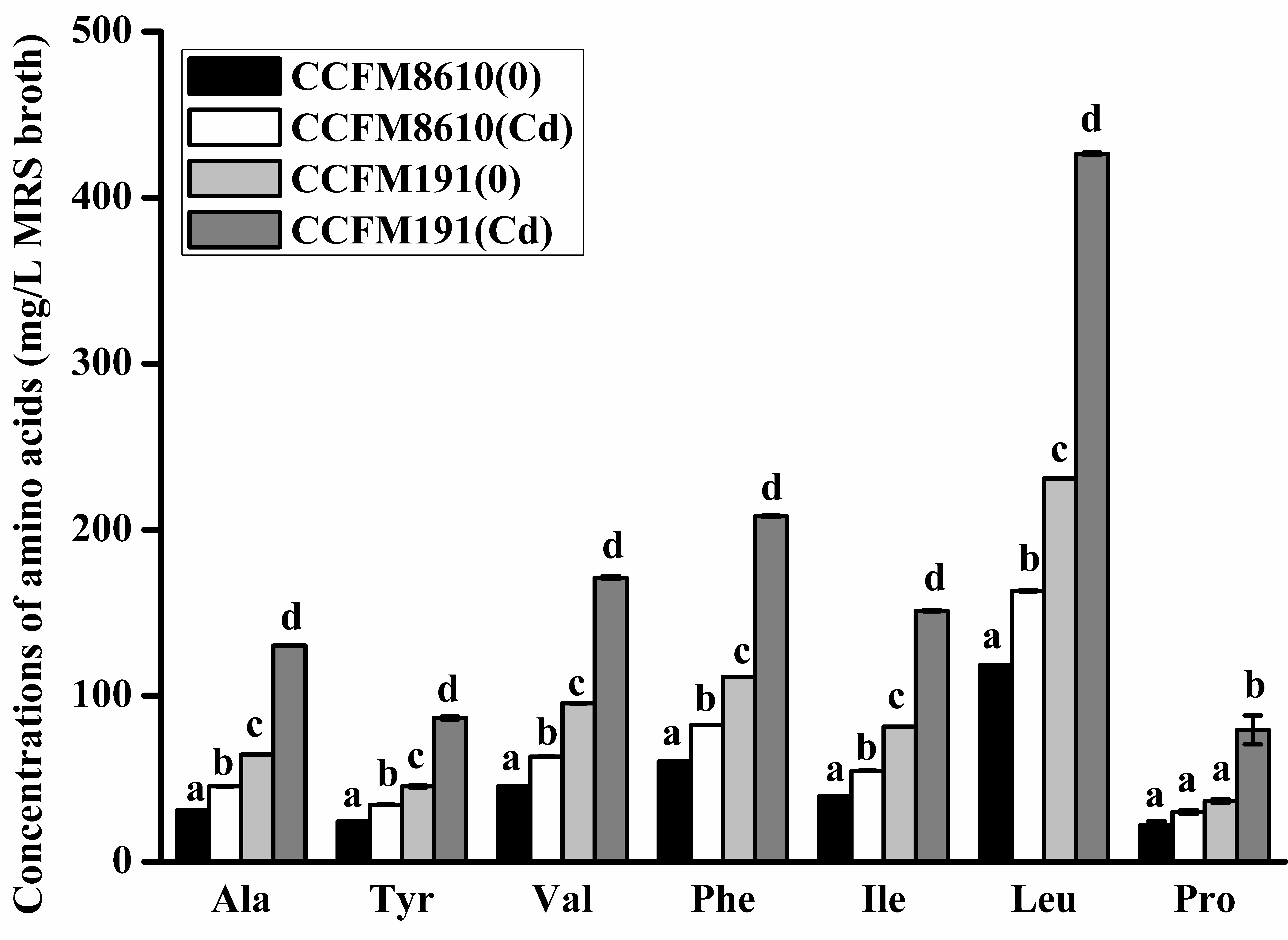


Figure S11


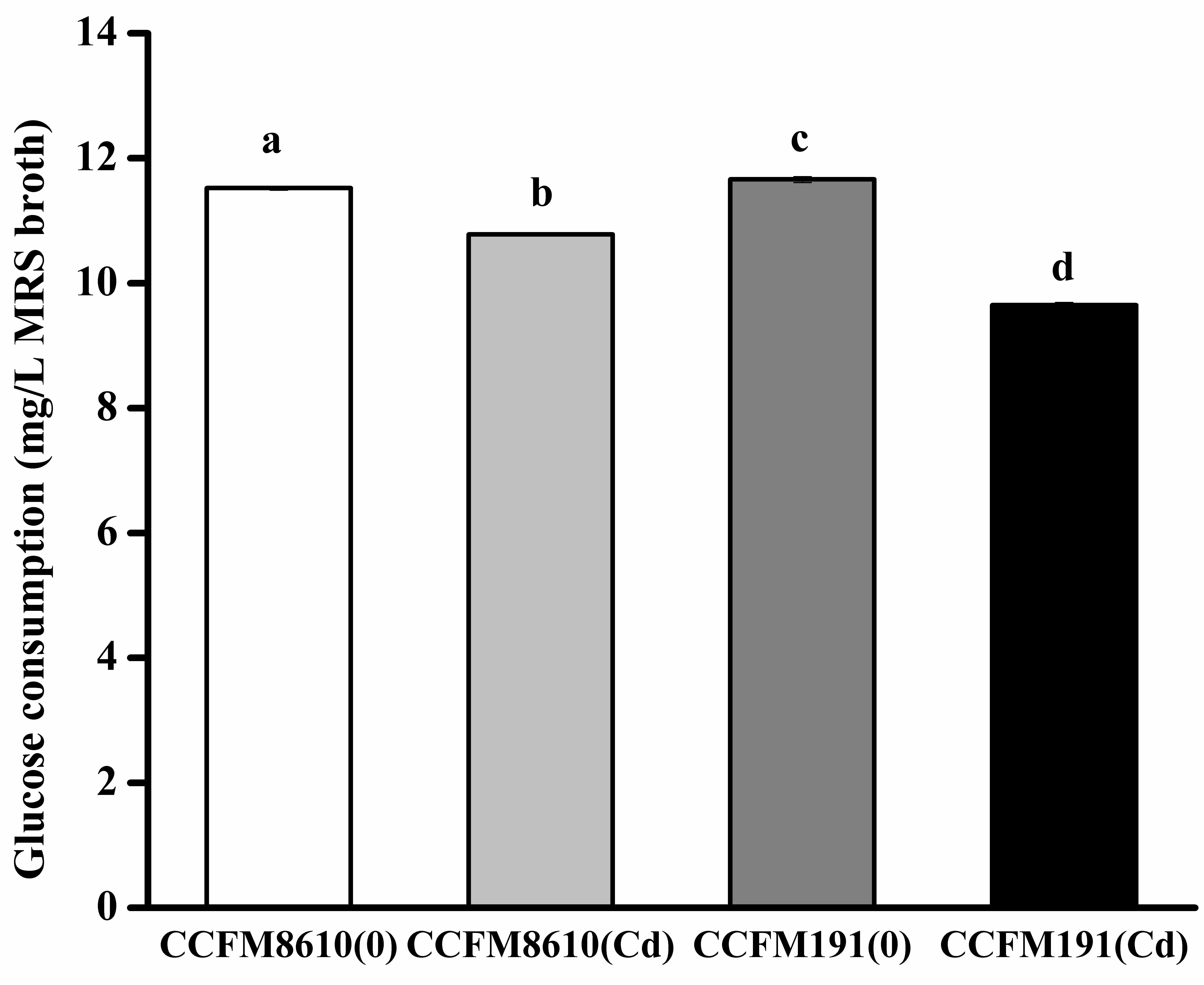

Supplement: Supplementary file 1 — Supplementary Info [file 41598_2017_1180_MOESM1_ESM.doc]
